# Comparative Evaluation of Cross-Sectional Geometric Feature Extraction Algorithms for LiDAR-Based Inclination Detection of Lattice Steel Towers

**DOI:** 10.3390/s26175558

**Published:** 2026-09-01

**Authors:** Mingduan Zhou, Guanxiu Wu, Lu Qin, Shufa Li

**Affiliations:** School of Geomatics and Urban Spatial Informatics, Beijing University of Civil Engineering and Architecture, Beijing 102616, China; 2108160225010@stu.bucea.edu.cn (G.W.); 2108570424058@stu.bucea.edu.cn (L.Q.); 2108570425026@stu.bucea.edu.cn (S.L.)

**Keywords:** LiDAR point cloud, inclination detection, RANSAC algorithm, Marching Square algorithm, cross-sectional feature extraction, lattice steel tower

## Abstract

**Highlights:**

**What are the main findings?**

- A unified evaluation framework integrating cross-sectional slicing, feature extraction, and axis reconstruction was proposed for LiDAR-based inclination detection of lattice steel towers. The framework enables a comparative evaluation of the performance of the RANSAC and Marching Square algorithms for cross-sectional geometric feature extraction under open-frame structural conditions.
- An in-service 110 kV overhead transmission tower was selected as the experimental object. Under different cross-sectional slicing strategies, the inclination values obtained by the RANSAC algorithm ranged from 8.52‰ to 8.82‰, with a range of 0.30‰ and a mean deviation of 0.12‰. In comparison, the Marching Square algorithm exhibited a range of 1.00‰ and a mean deviation of 0.41‰. The RANSAC algorithm provided more consistent inclination estimation than the Marching Square algorithm, whereas the Marching Square algorithm achieved better preservation of cross-sectional contour representation.

**What are the implications of the main findings?**

- The proposed framework enables objective comparison and selection of feature extraction algorithms for point cloud-based structural inspection.
- The results provide technical support for reliable inclination assessment and intelligent operation and maintenance of complex lattice steel structures.

**Abstract:**

Non-contact inclination detection based on LiDAR point clouds has become an effective approach for structural condition assessment. However, due to measurement noise, lattice structural characteristics, and discontinuous point distributions, cross-sectional point clouds of lattice steel towers often contain outliers, local missing regions, and irregular boundaries, which may affect the reliability of extracted geometric features. This study establishes a comparative framework to investigate the influence of cross-sectional feature extraction algorithms on LiDAR-based inclination detection of lattice steel towers. The universality and performance of the RANSAC and Marching Square algorithms were systematically evaluated using a 110 kV overhead transmission line operating tower. Terrestrial laser scanning was employed to acquire the point cloud data. The initial registration results were subsequently further optimized through initial point cloud registration and multi-station adjustment. Four cross-sectional slicing schemes were designed, and the two algorithms were independently applied to extract cross-sectional geometric features and calculate centroid coordinates. The tower inclination was then determined by fitting the spatial distribution of centroid points. Experimental results demonstrated that both algorithms successfully extracted cross-sectional features and achieved reliable inclination detection results, with all inclination ratios satisfying the requirement specified in DL/T 741—2019 (Code of Practice for Operation of Overhead Transmission Lines). The RANSAC-based method produced inclination ratios ranging from 8.52‰ to 8.82‰, with a variation range of 0.30‰ and a mean deviation of 0.12‰. In comparison, the Marching Square-based method showed a larger variation range of 1.00‰ and a mean deviation of 0.41‰. The results indicate that RANSAC provides better robustness against point cloud noise, local data gaps, and boundary irregularities due to its inlier–outlier discrimination capability, whereas Marching Square exhibits advantages in preserving continuous contour representations when point cloud distributions are relatively complete. This study provides practical insights into the selection and optimization of cross-sectional feature extraction algorithms for LiDAR-based inclination assessment of lattice steel towers.

## 1. Introduction

Lattice steel towers have been widely applied in the fields of electric power transmission [1], communication broadcasting [2], construction engineering [3], and urban landmarks [4] due to their structural characteristics of lightweight design, open-frame configuration, and high wind resistance. However, under the combined effects of environmental corrosion, ground settlement, and long-term service operation, tower structures are prone to deformation phenomena such as inclination and torsion, which may even lead to structural collapse in severe cases [5,6]. Among these deformation types, inclination deformation can alter the structural load-transfer path and reduce the positioning accuracy of equipment installed at the tower top. Therefore, the development of robust and reliable inclination measurement methods is of great significance for ensuring the operational safety, extending the service life, and optimizing maintenance strategies of lattice steel towers [7].

Traditional measurement methods mainly rely on geodetic techniques such as Global Navigation Satellite System (GNSS), total stations [8], theodolites [9], and precise leveling. By periodically measuring the three-dimensional coordinates or elevations of monitoring points, these methods can provide information on structural deformation. For example, Zhou et al. [10] employed GNSS technology to determine the verticality of a tower crane, while Song et al. [11] used the plane mirror method to measure the inclination rate of a transmission tower. However, traditional geodetic methods generally suffer from low measurement efficiency, high labor intensity, and susceptibility to human-induced errors. Moreover, the limited number of monitoring points makes it difficult to comprehensively characterize the overall deformation of complex structures. In recent years, with the advancement of measurement technologies, tower inclination detection has gradually evolved toward emerging technical approaches represented by sensor-based monitoring, computer vision, and LiDAR point cloud-based measurement. According to whether physical contact with the target structure is required, these methods can be generally classified into contact-based and non-contact-based approaches.

In terms of contact-based measurement, existing studies have mainly focused on the installation of inclination sensors, fiber Bragg grating sensors, or inertial measurement units on tower structures to achieve real-time acquisition of structural attitude information (GAO et al. [12]; Luo et al. [13]; Gemayel et al. [14]; Ngabo et al. [15]; Zhang et al. [16]). Such methods are capable of enabling long-term remote monitoring and have achieved relatively high measurement accuracy under specific conditions. However, contact-based approaches require the installation of sensing devices directly onto tower bodies, resulting in relatively high installation and maintenance costs. Moreover, sensor performance can be affected by environmental temperature variations, electromagnetic interference, and other external factors, leading to data drift or measurement uncertainties. In addition, the requirement for individual sensor deployment on each tower significantly limits the efficiency of large-scale inspection applications.

To overcome the limitations of contact-based approaches, non-contact measurement methods have gradually become a research focus, mainly including image-based visual measurement and three-dimensional reconstruction methods based on point clouds. For vision-based measurement, Yang et al. [17], Feng et al. [18], Ge et al. [19], and Chen et al. [20] conducted inclination detection studies of buildings and transmission towers using monocular or binocular vision systems. Structural side edges or central axes were extracted through approaches such as manual feature marking, image rectification, and deep learning-based semantic segmentation, followed by inclination parameter estimation. Liu et al. [21] and Qiao et al. [22] further integrated unmanned aerial vehicle imagery with deep learning techniques to expand the application scenarios of visual inspection methods. However, vision-based methods typically rely on stable illumination conditions and clear structural textures, and their applicability is limited for lattice towers with complex structures and severe occlusion. In contrast, three-dimensional point cloud-based measurement methods can directly acquire the spatial geometric information of structures. Their deformation analysis can generally be classified according to point cloud modeling and geometric information extraction strategies into different categories, including methods based on geometric difference comparison among multi-temporal point clouds and methods based on geometric modeling or structural feature extraction from point clouds [23]. The former registers point clouds acquired at different times and compares spatial changes in corresponding points, surfaces, or geometric features to monitor structural deformation or displacement. The latter establishes the relationship between structural geometric information and deformation parameters through point cloud stratification, cross-sectional analysis, geometric model fitting, or feature parameter extraction. This classification reflects the technical characteristics of different deformation detection methods from the perspectives of point cloud data processing and geometric modeling. Among methods based on multi-temporal point cloud comparison, Zhao et al. [24] monitored tower deformation through multi-temporal point cloud registration and inferred inclination-induced displacement by comparing changes in corresponding point positions between different observation periods. Among methods based on geometric modeling and structural feature extraction, Xie et al. [25], Shen et al. [26], Liu et al. [27], and Wu et al. [28] acquired tower point clouds using terrestrial three-dimensional laser scanning and extracted cross-sectional or structural geometric features using methods such as layered slicing, Alpha Shapes, and least-squares fitting to calculate inclination. Huang et al. [29], Chen et al. [30], Li et al. [31], and Xu et al. [32], in contrast, used unmanned aerial vehicle LiDAR point clouds and employed methods such as Random Sample Consensus (RANSAC) fitting, DBSCAN clustering, and minimum bounding rectangles to extract tower structural information and analyze inclination conditions. From the perspective of point cloud acquisition platforms, these studies further encompass different data acquisition approaches, including terrestrial laser scanning and UAV LiDAR, demonstrating the strong applicability of point cloud-based techniques to deformation detection of complex tall structures. Although these studies have successfully achieved tower inclination detection based on point cloud data, existing research has mainly concentrated on extracting the overall structural axis or principal member direction lines. A systematic investigation into the influence of different cross-sectional feature extraction algorithms on inclination detection results and their applicability under complex point cloud conditions remains insufficient.

Cross-sectional feature extraction represents a critical step in the point cloud-based inclination detection workflow, as the selection of extraction algorithms directly affects the accuracy of central axis fitting and the reliability of final inclination parameters. Currently, among studies focusing on point cloud cross-sectional feature extraction, the RANSAC algorithm and the Marching Square algorithm are two representative approaches, each exhibiting distinct advantages in different application scenarios. The RANSAC algorithm, proposed by Fischler and Bolles [33], is a robust model estimation method based on random sampling and consensus evaluation mechanisms, which enables reliable parameter estimation in the presence of substantial abnormal observations. Subsequently, Schnabel et al. [34] introduced RANSAC into the field of three-dimensional point cloud shape detection, enabling the automatic extraction of fundamental geometric primitives, including planes, spheres, and cylinders, and providing an effective technical approach for geometric feature analysis in complex environments. For tower structure inspection, Huang et al. [29] extracted structural member lines using RANSAC fitting and generated the central axis direction of the tower. Chen et al. [30] applied RANSAC-based line fitting to layered tower point clouds and achieved inclination measurement through inclination vector calculation. Furthermore, RANSAC has been extensively applied in tasks such as building facade extraction, tunnel cross-sectional analysis, and structural line extraction of bridges. For cross-sectional contour extraction, RANSAC repeatedly performs plane model fitting and inlier evaluation on sliced point clouds, thereby effectively removing outlier noise and providing strong applicability for lattice tower cross-sectional point clouds containing local missing regions and measurement disturbances. The Marching Cubes algorithm, proposed by Lorensen and Cline [35], was originally developed for isosurface extraction from three-dimensional scalar fields. Subsequently, based on regular grid traversal and lookup-table strategies, its two-dimensional extension, known as the Marching Squares algorithm, was developed for contour extraction from two-dimensional scalar fields. This algorithm determines contour intersection points according to the states of individual grid cells and generates continuous closed boundaries. In point cloud processing, the Marching Square algorithm has been widely applied in tasks such as building footprint reconstruction, terrain contour extraction, and structural cross-sectional profile reconstruction. Zhou et al. [36,37] employed the Marching Square algorithm for tower crane verticality inspection. Through regular grid interpolation, this algorithm can achieve sub-grid accuracy in contour localization and provides effective geometric representation for cross-sectional point clouds with continuous boundaries. However, the performance of the Marching Square algorithm highly depends on the completeness of point cloud distributions, and local voids or sparse point regions may result in contour discontinuities or geometric distortions.

Considering that RANSAC and Marching Square algorithms emphasize noise robustness and geometric contour preservation, respectively, the two approaches exhibit different advantages in cross-sectional feature extraction of lattice steel towers. Therefore, this study proposes a comparative framework for cross-sectional feature extraction algorithms in LiDAR-based inclination detection of lattice steel towers, and the effectiveness and reliability of the proposed framework are validated through field experiments. The main contributions of this study are summarized as follows. (1) A unified framework for cross-sectional feature extraction and inclination detection of lattice steel towers was established. Considering the discrete distribution, local data gaps, and noise interference commonly existing in lattice tower point clouds, a complete processing workflow was developed, including point cloud preprocessing, cross-sectional slicing, cross-sectional feature extraction, centroid calculation, axis fitting, and inclination parameter estimation. This framework provides a unified data processing basis for performance comparison among different feature extraction algorithms. (2) The integration and comparative analysis of two representative cross-sectional feature extraction algorithms, namely RANSAC and Marching Square, were achieved. According to the geometric feature extraction requirements of lattice tower cross sections, the RANSAC algorithm based on random consensus estimation was employed to obtain cross-sectional geometric information, while the Marching Square algorithm based on contour tracing was adopted for comparative analysis. (3) A stability evaluation and comparative analysis mechanism for inclination detection under different sampling scales was established. Multiple cross-sectional slicing schemes were designed to investigate the variation characteristics of inclination detection results obtained by the two algorithms under different spatial sampling conditions. Comprehensive evaluations were conducted from perspectives including inclination consistency, result dispersion, and adaptability to noise and local missing data, thereby revealing the applicability characteristics of different algorithms under complex point cloud environments. (4) Field experiments and engineering applicability evaluations were conducted. An operating overhead transmission line tower was selected as the experimental object, and the RANSAC and Marching Square algorithms were tested and comparatively analyzed in a real-world environment. The feasibility of the proposed algorithm comparison framework for practical inclination detection of lattice steel towers was verified, providing references for selecting appropriate point cloud feature extraction methods for similar high-rise steel structures.

The remainder of this paper is organized as follows. Section 2 introduces the methods and materials adopted in this study. Section 3 describes the experimental design, data preprocessing procedures, and cross-sectional slicing schemes in detail. Section 4 presents the inclination results. Section 5 provides a discussion and analysis of the obtained results. Finally, Section 6 summarizes the main conclusions of this study and discusses future research directions.

## 2. Methods

**Figure 1:**
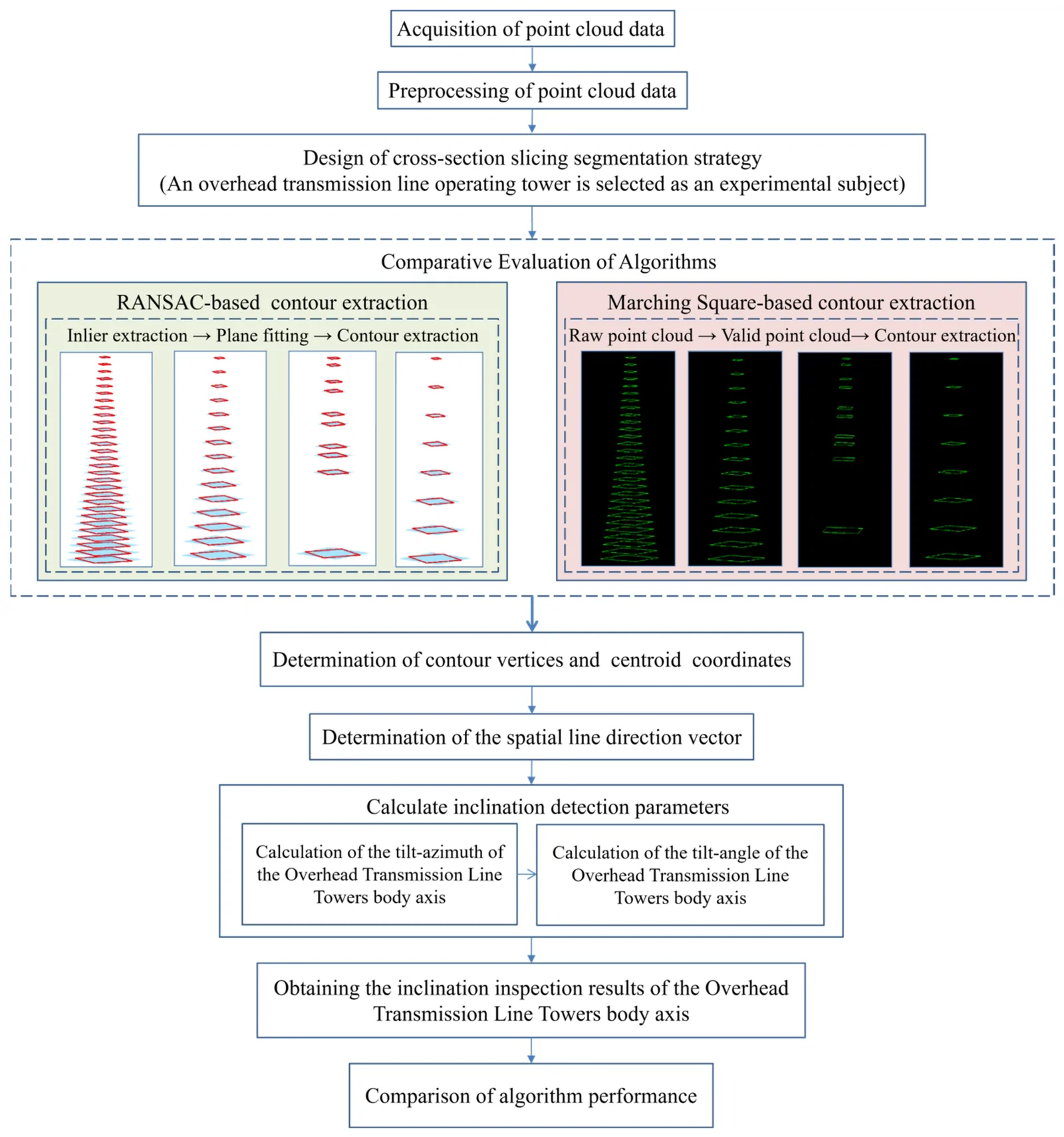
Comparative framework for inclination detection algorithms.

The comparison framework for comparative evaluation of cross-sectional geometric feature extraction algorithms for LiDAR-based inclination detection of lattice steel towers is designed as follows. First, point cloud data of the lattice steel tower are acquired using a terrestrial laser scanner. Point cloud registration is performed using no fewer than four corresponding feature points, followed by point cloud segmentation based on the spatial distribution characteristics of the main tower body and other structural components to obtain a high-precision point cloud of the tower body. Subsequently, cross-sectional point clouds at different elevations are extracted according to the predefined cross-sectional slicing schemes. The RANSAC and Marching Squares algorithms are then separately employed to extract the geometric features of the cross-sections, followed by calculation of the centroid coordinates of each cross-section. The cross-sectional centroids distributed along the height direction are fitted using a spatial straight line to obtain the direction vector of the tower central axis. Finally, in the station-centered spatial coordinate system O-XYZ (a unified station-centric spatial coordinate system O-XYZ was established based on the first scanning station. The origin O was defined at the center of the terrestrial laser scanner instrument at the first scanning station, the *Z*-axis was aligned with the plumb direction, the *X*-axis was defined along the initial horizontal orientation of the scanner at the first station, and the *Y*-axis was determined according to the right-hand rule.), the inclination azimuth, inclination angle, and inclination ratio are calculated based on the geometric relationships between the central-axis direction vector, the horizontal reference direction, and the vertical direction. The stability and applicability of the two cross-sectional feature extraction algorithms are evaluated by comparing the detection results obtained under different cross-sectional sampling schemes. The overall workflow is illustrated in Figure 1.

### 2.1. High-Precision Point Cloud Data Acquisition of Lattice Steel Towers

Original point cloud data collection. The terrestrial laser scanner is set up at a suitable location with a clear line of sight and stable ground facing the latticed steel tower. Scanning measurements are performed to acquire spatial distances and horizontal and vertical deflection angle data of the latticed steel tower surface, and the point cloud data are obtained in the station-centered spatial coordinate system (O-XYZ) [38], as expressed in Equation (1).

(1) $$P=(x_{i},y_{i},z_{i})=(S\cdot \cos\theta \cdot \cos\alpha ,S\cdot \cos\theta \cdot \sin\alpha ,S\cdot \sin\theta )$$

where S represents the distance measured by the terrestrial laser scanner to the latticed steel tower surface; α represents the horizontal deflection angle; and θ denotes the vertical deflection angle.

1. High-precision effective point cloud data acquisition. The first scanning station was used as the reference station, and scanning stations 2–6 were individually registered to the first scanning station. Four groups of feature points that were non-coplanar in three-dimensional space and differed in both elevation and horizontal position were selected for initial registration, and the initial registration results were further optimized through multi-station adjustment. All registered point clouds were ultimately transformed into the station-centered spatial coordinate system O-XYZ.

### 2.2. Cross-Sectional Feature Extraction

#### 2.2.1. Cross-Section Slicing Segmentation Strategy

1. Cross-sectional slicing. In the station-centered spatial coordinate system (O-XYZ), the preprocessed latticed steel tower body point cloud is horizontally sliced along the *z*-axis at Δh height intervals to obtain sliced point cloud data. The spatial range of the cross section can be represented as shown in Equation (2):
  (2) $$\Delta z=[z_{j}-\frac{\Delta h}{2},z_{j}+\frac{\Delta h}{2}]$$
  where $z_{j}$ represents the elevation of the j−th cross-sectional slice, j=1,2,⋯,m, and m denotes the total number of cross-sectional slices. The point cloud data located within the corresponding elevation range are assigned to the j−th cross section, and the corresponding point set can be expressed as shown in Equation (3):
  (3) $$P_{j}=\left\{p_{i}=(x_{i},y_{i},z_{i})\left|z_{j}-\frac{\Delta h}{2}\leq z_{i}\leq z_{j}+\frac{\Delta h}{2}\right.\right\}$$
  where $P_{j}$ represents the point cloud dataset of the j−th slice; $p_{i}$ denotes the i−th three-dimensional point within the j−th slice, where $i=1,2,\cdots ,n_{j}$; and $n_{j}$ indicates the number of point cloud points contained in the j−th slice.
2. The cross-sectional slicing interval $z_{j}$ should be determined by considering the dimensions of the lattice steel tower and the point cloud density to achieve a balance between spatial sampling density and computational efficiency. The slice thickness Δh should be determined according to the point cloud density and measurement accuracy of the terrestrial laser scanner.

#### 2.2.2. Cross-Sectional Contour Extraction of Latticed Steel Towers

Cross-sectional contour extraction. To investigate the influence of different cross-sectional geometric feature extraction algorithms on the inclination detection results of lattice steel towers, the RANSAC and Marching Square algorithms were separately applied to the cross-sectional slices. Both methods first transform the cross-sectional point clouds at the selected elevations into a local two-dimensional coordinate system corresponding to the cross-sectional plane. The RANSAC algorithm identifies valid points through robust plane fitting, whereas the Marching Squares algorithm constructs a two-dimensional distance scalar field from the projected point cloud and extracts the cross-sectional contour from the scalar field. Subsequently, the extracted closed contours are used to calculate the cross-sectional centroids using an area-weighted method, and the resulting centroid coordinates are used for central-axis reconstruction and inclination estimation [39,40].

**Figure 2:**
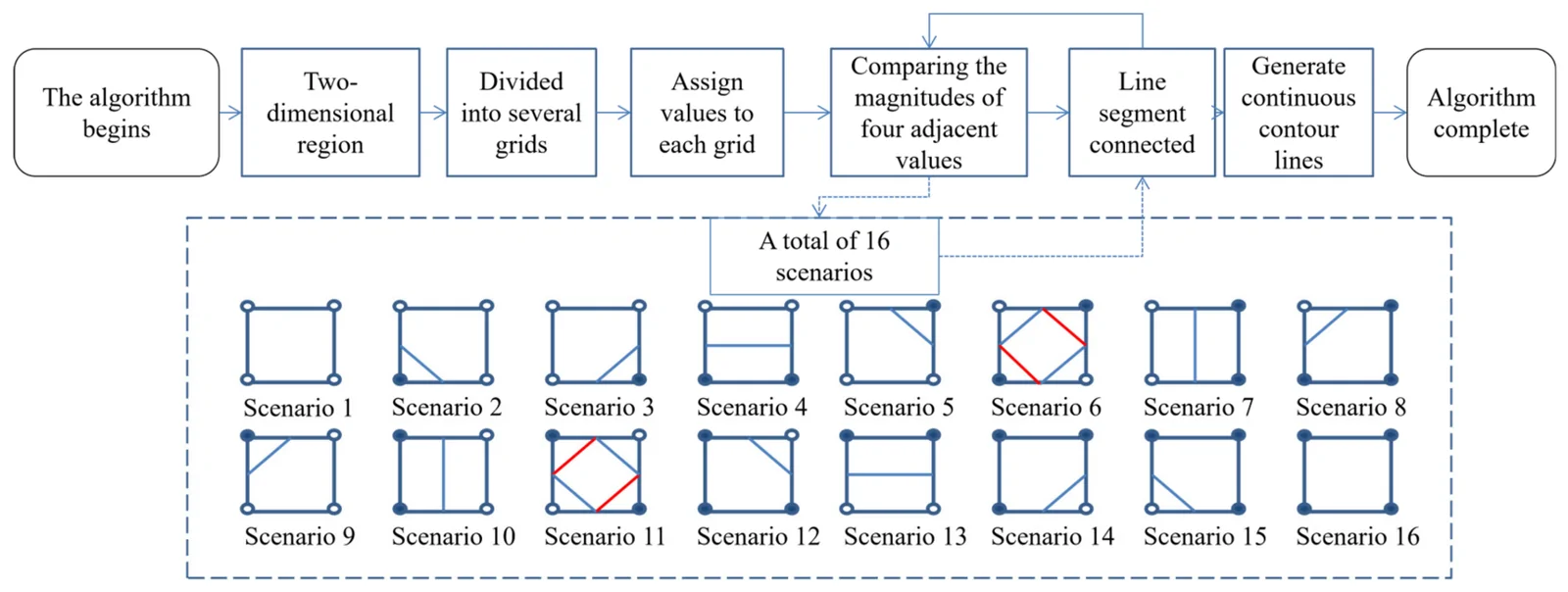
Workflow of the Marching Square-based cross-sectional contour extraction method.

- Cross-Sectional Feature Extraction Based on the RANSAC Algorithm. For each cross-sectional slice, robust plane fitting is performed using the RANSAC algorithm to identify a valid point set consistent with the geometric structure of the cross-section. The RANSAC algorithm randomly selects the minimum sample set satisfying the requirements for plane fitting from the cross-sectional slice to construct a candidate cross-sectional plane, and calculates the orthogonal distances from the remaining points to the candidate plane. Based on a predefined distance threshold, points satisfying the threshold criterion are classified as inliers, while the remaining points are classified as outliers. Through repeated random sampling and model iterations, the candidate planes are evaluated based on the number of inliers and the model support, and the plane with the largest number of inliers is selected as the final fitted plane. The resulting inlier set is used as the valid point set for subsequent cross-sectional geometric feature extraction. Boundary feature points are determined from the projection extrema of the in-plane points along two orthogonal coordinate directions and connected according to their spatial order to form an approximately closed cross-sectional contour for subsequent area-weighted centroid calculation. Finally, the obtained cross-sectional centroid coordinates are used for central-axis reconstruction and inclination parameter estimation. The specific algorithm steps are as follows:

1. First, $s_{e}$ non-collinear points are randomly selected from each cross-sectional sliced point cloud to form a minimal sample subset, uniquely determining a plane model, as expressed in Equation (4).
  (4) $$E=\left\{a_{1},a_{2},\ldots ,a_{s_{e}}\right\}$$
  where E denotes the minimal sample subset selected from the cross-sectional point cloud, $a_{i}$ represents the i−th selected point in this subset, and $s_{e}$ is the number of points contained in E. For plane fitting, three non-collinear points are required; hence $S_{e}=3$.
2. Secondly, based on this sample subset, the candidate planar model is constructed as shown in Equation (5).
  (5) Ax+By+Cz+D=0
  where $\left(A,B,C\right)$ represents the normal vector coefficients of the candidate plane, and D denotes the constant coefficient of the plane equation.
3. Subsequently, the orthogonal distances from all points in the cross-sectional sliced point cloud to the candidate plane are computed to evaluate the consistency of the candidate model, as shown in Equation (6).
  (6) $$d_{r}=\frac{\left|Ax_{r}+By_{r}+Cz_{r}+D\right|}{\sqrt{A^{2}+B^{2}+C^{2}}}$$
  where $\left(x_{r},y_{r},z_{r}\right)$ denotes the three-dimensional coordinates of the r−th point, and $d_{r}$ represents the distance from this point to the candidate plane. A distance threshold $d_{t}$ is defined; when $d_{r}\leq d_{t}$, the point is classified as an inlier; otherwise, it is treated as an outlier.
4. Accordingly, the inlier set corresponding to the candidate model is obtained, as shown in Equation (7).
  (7) $$H=\left\{p_{r}\left|d_{r}\leq d_{t}\right.\right\}$$
  where $p_{r}=(x_{r},y_{r},z_{r})$ denotes the r−th point in the cross-sectional point cloud, and H represents the inlier set identified by the candidate plane model.
5. Through multiple iterations, the model parameters corresponding to the maximum number of inliers are continuously updated. When the iteration termination criterion is satisfied, the optimal model is selected as the final result, as shown in Equation (8).
  (8) $$N=\frac{\log\left(1-p\right)}{\log\left(1-w^{s}\right)}$$
  where N denotes the maximum number of iterations, w represents the proportion of inliers in the point cloud, and p is the desired confidence probability.

- Contour Extraction Based on the Marching Square Algorithm. The Marching Square algorithm is a contour extraction method based on a two-dimensional regular grid. Its core principle is to determine the intersection points between the target region boundary and the grid boundaries by analyzing the scalar field distribution within local grid cells, and to then connect all local contour segments to obtain a continuous boundary. The detailed workflow is presented in Figure 2.

The corresponding implementation steps based on the workflow shown in Figure 2 are as follows:

1. First, the cross-sectional point cloud is projected onto the horizontal XY plane of the station-centered spatial coordinate system O-XYZ to obtain a two-dimensional point cloud. The projected point cloud set is expressed as shown in Equation (9).
  (9) $$P=\left\{(x_{i},y_{i}),i=1,2,\ldots ,n\right\}$$
  where P represents a two-dimensional point set composed of n coordinate vectors, n denotes the number of points in the cross-sectional point cloud, and $\left(x_{i},y_{i}\right)$ represents the coordinates of the i−th point on the two-dimensional projection plane.
2. Secondly, a two-dimensional regular grid is generated within the spatial extent of the projected point cloud, as shown in Equation (10).
  (10) $$G=\left\{g_{ij}\left|i=1,\ldots m,\;j=1,\ldots ,k\right.\right\}$$
  where $g_{ij}$ denotes the grid node at the i−th row and j−th column of the two-dimensional regular grid, which is used as the discrete sampling position for constructing the distance scalar field. m and k indicate the numbers of grid nodes along the x− and y− directions, respectively. The grid cell size (i.e., spatial resolution) is determined based on the point cloud density and the structural scale of the tower body. It is generally set to 1–2 times the average point spacing of the sliced point cloud to ensure a balance between contour extraction accuracy and computational efficiency.
3. Subsequently, the discrete cross-sectional point cloud is converted into a continuous two-dimensional scalar field. The distance field is employed as the input for the Marching Square algorithm, where the scalar value of each grid node is defined as the Euclidean distance to the nearest point cloud point, as shown in Equation (11).
  (11) $$D\left(g_{ij}\right)=\min\left\|g_{ij}-p_{l}\right\|$$
  where $D\left(g_{ij}\right)$ denotes the distance field value of the grid node $g_{ij}$ and $p_{l}$ represents an arbitrary point in the projected cross-sectional point cloud. Accordingly, each grid node is assigned a distance value, resulting in the generation of a continuously varying two-dimensional distance field.
4. After the two-dimensional distance scalar field is obtained, an iso-value threshold T is introduced to separate the cross-sectional region from the background. Grid nodes satisfying $D\left(g_{ij}\right)\leq T$ are classified as cross-sectional regions, whereas the remaining nodes are assigned to the background region.
5. The Marching Square algorithm subsequently traverses the two-dimensional regular grid and determines the states of the four vertices in each 2×2 grid cell. Based on the relationship between the vertex values and the iso-value threshold, each grid cell is assigned to one of 16 possible topological configurations. The contour intersection locations are obtained using a predefined lookup table, and the coordinates of these intersection points are calculated by linear interpolation, as shown in Equation (12).
  (12) $$P_{c}=P_{1}+\frac{T-D_{1}}{D_{2}-D_{1}}(p_{2}-p_{1})$$
  where $P_{c}$ denotes the coordinate of the contour-grid intersection point. $p_{1}$ and $p_{2}$ represent two adjacent grid nodes on the corresponding edge, while $D_{1}$ and $D_{2}$ indicate their associated scalar values, respectively.
6. By connecting the contour segments generated by all grid cells, a complete closed cross-sectional contour is reconstructed. Due to occlusions, limited scanning viewpoints, and gaps between structural members during terrestrial laser scanner data acquisition, multiple closed contours may exist in the extracted cross-sectional point cloud. To reduce the influence of internal voids and locally missing regions on centroid estimation, an area-based contour selection strategy is introduced. The contour with the largest enclosed area is retained as the valid external boundary of the cross section.

### 2.3. Central Axis Reconstruction and Inclination Estimation

#### 2.3.1. Area-Weighted Centroid Calculation

To reduce the influence of different cross-sectional feature extraction algorithms on centroid estimation, an area-weighted method is employed in this study to calculate the centroids of the contours extracted by the RANSAC and Marching Square algorithms. The specific calculation procedures are described as follows:

1. First, assume that the extracted closed cross-sectional contour consists of n vertices, and their two-dimensional coordinates are represented, as shown in Equation (13).
  (13) $$p_{i}=(x_{i},y_{i}),i=1,2,\cdots ,n$$
  where the contour vertices are sequentially arranged in either a clockwise or counterclockwise direction and satisfy the $\left(x_{n+1},y_{n+1})=(x_{1},y_{1}\right)$.
2. Second, the area of the contour is calculated. The signed area enclosed by the contour can be obtained using the cross-product formulation based on the vertex coordinates, as expressed in Equation (14).
  (14) $$A=\frac{1}{2}\sum_{i=1}^{n}\left(x_{i}y_{i+1}-x_{i+1}y_{i}\right)$$
  where A represents the signed area enclosed by the contour, and $x_{i}y_{i+1}-x_{i+1}y_{i}$ denotes the oriented area contribution generated by the adjacent vertices $p_{i}$ and $p_{i+1}$. The value of A is positive when the vertices are ordered counterclockwise and negative when they are ordered clockwise.
3. Subsequently, the two-dimensional centroid coordinates of the contour are determined using the centroid formula based on the calculated contour area, as expressed in Equation (15).
  (15) $$\begin{array}{l} x_{j}=\frac{1}{6A}\sum_{i=1}^{n}\left(x_{i}+x_{i+1}\right)(x_{i}y_{i+1}-x_{i+1}y_{i})\\ y_{j}=\frac{1}{6A}\sum_{i=1}^{n}\left(y_{i}+y_{i+1}\right)(x_{i}y_{i+1}-x_{i+1}y_{i}) \end{array}$$
  where $\left(x_{j},y_{j}\right)$ denotes the centroid coordinates of the cross-sectional contour in the local two-dimensional coordinate system, and j=1,2,⋯,m, m represents the total number of vertices of the contour. The coefficient $\frac{1}{6A}$ is a normalization factor introduced to transform the first-order area moments into the centroid coordinates.
4. Finally, the two-dimensional centroid coordinates are transformed back into the three-dimensional coordinate system. The z-coordinate of the contour centroid is calculated as the arithmetic mean of the z-coordinates of all vertices constituting the contour. Accordingly, the three-dimensional centroid coordinates of the cross-sectional contour are obtained, as expressed in Equation (16).
  (16) $$o_{j}=(x_{j},y_{j},z_{j})=\left(\frac{1}{6A}\sum_{i=1}^{n}\left(x_{i}+x_{i+1}\right)(x_{i}y_{i+1}-x_{i+1}y_{i}),\frac{1}{6A}\sum_{i=1}^{n}\left(y_{i}+y_{i+1}\right)(x_{i}y_{i+1}-x_{i+1}y_{i}),\bar{z}\right)$$
5. Therefore, the centroids of all extracted contours can be obtained, and these centroid points are represented as a set in Equation (17).
  (17) $$O=\left\{o_{1},o_{2},\cdots ,o_{j},\cdots ,o_{m}\right\}$$
  where O represents the set of centroids of all contours.

#### 2.3.2. Determination of the Spatial Line Equation and Direction Vector of Lattice Steel Tower Structures Central Axis

The spatial central axis of a lattice steel tower is defined as a three-dimensional straight line representing the longitudinal geometric centerline of the tower structure. It is determined from the spatial distribution of the geometric centroids extracted from multiple cross-sectional slices. Each cross-sectional centroid represents the geometric center of the corresponding section, and the central axis is obtained by fitting a spatial straight line to the set of centroid points. Because the tower may exhibit inclination, the reconstructed central axis does not necessarily coincide with the global vertical axis. Its direction is represented by a unit direction vector, which is subsequently used to calculate the inclination parameters of the tower.

The spatial central axis can be expressed as

(18) $$L(\lambda )=R_{0}+\lambda d_{0}$$

where $R_{0}={\left(R_{0x},R_{0y},R_{0z}\right)}^{T}$ denotes a reference point on the central axis, $d_{0}={\left(d_{x},d_{y},d_{z}\right)}^{T}$ denotes the unit direction vector of the central axis, λ is a scalar parameter, and $\left\|d_{0}\right\|$ = 1.

**Figure 3:**
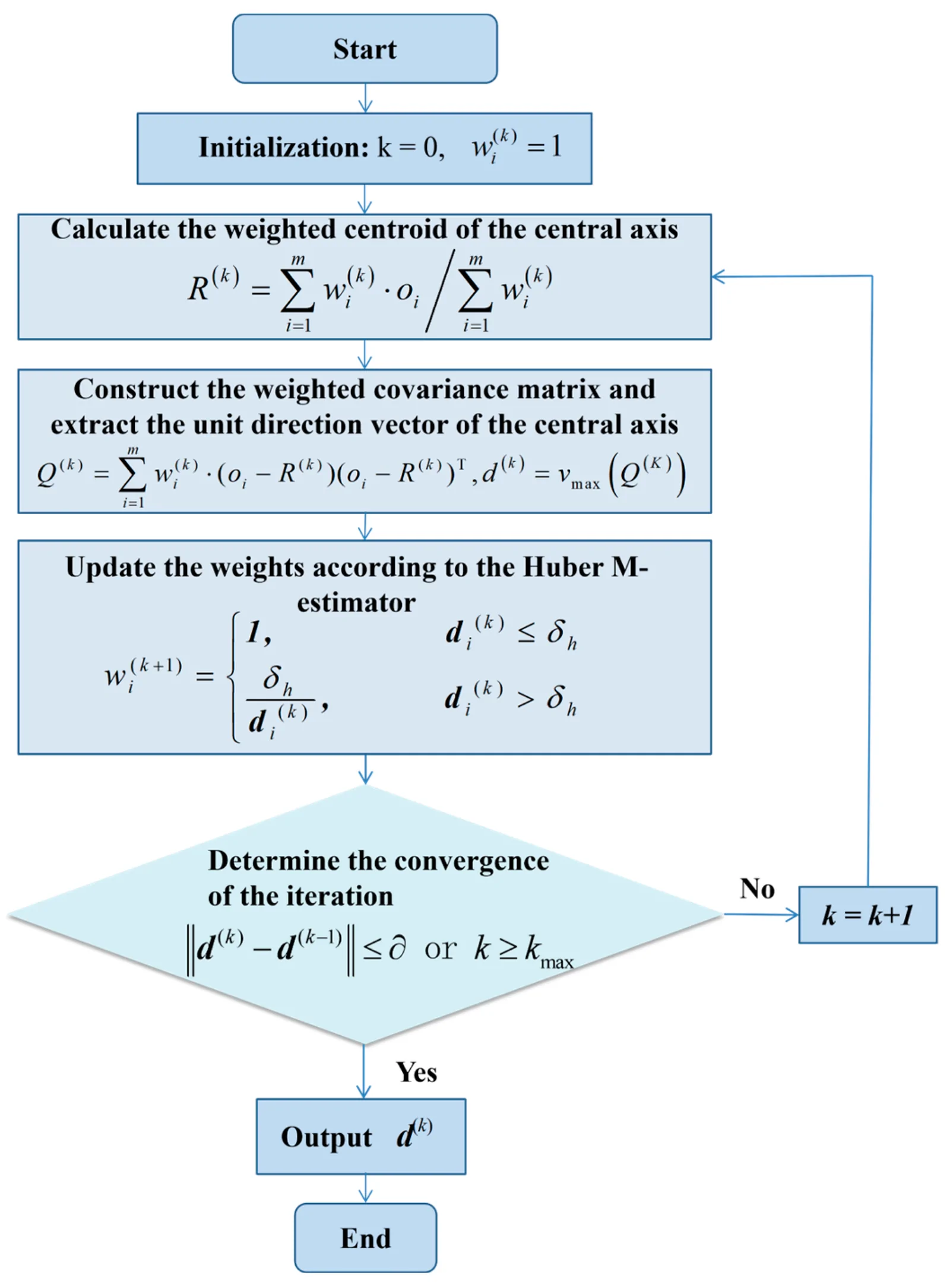
Flowchart of the unit direction vector determination algorithm.

Based on the contour given in Equation (17), and considering that the Iterative Reweighted Least Squares (IRLS) method can effectively suppress the influence of outliers and local noise on fitting results, this study adopts the IRLS algorithm to determine the unit direction vector of the central axis of the lattice tower structures [41]. The detailed procedure is illustrated in Figure 3.

The corresponding implementation steps based on the algorithm flow shown in Figure 3 are as follows:

1. Computation of the Weighted Centroid of the Central Axis. At the initial iteration, equal weights are assigned to the contour centroid points of the cross-sectional contour, i.e., $w_{i}^{\left(0\right)}=1$. The weighted contour centroid is then computed using the updated weights, as expressed in Equation (19).
  (19) $$R^{\left(k\right)}=\sum_{i=1}^{m}w_{i}^{\left(k\right)}\cdot o_{i}/\sum_{i=1}^{m}w_{i}^{\left(k\right)}$$
  where $R^{\left(k\right)}$ denotes the weighted centroid of all contour centroids; $w_{i}^{\left(0\right)}=$1 represents the initial weight assigned to the i−th contour centroid; k denotes the iteration number; and k= 0 corresponds to the initial state.
2. Construction of the Weighted Covariance Matrix and Estimation of the Unit Direction Vector of the Central Axis. The weighted covariance matrix is constructed, followed by eigenvalue decomposition. The unit eigenvector corresponding to the largest eigenvalue is selected as the unit direction vector of the central axis, as expressed in Equation (20).
  (20) $$Q^{\left(k\right)}=\sum_{i=1}^{m}w_{i}^{\left(k\right)}\cdot (o_{i}-R^{\left(k\right)}){\left(o_{i}-R^{\left(k\right)}\right)}^{T},\;d^{\left(k\right)}=v_{\max}\left(Q^{\left(K\right)}\right)$$
  where $Q^{\left(k\right)}$ denotes the weighted covariance matrix of the set of contour centroid coordinates O in the current iteration; $d^{\left(k\right)}$ represents the unit direction vector of the central axis at the current iteration; and $v_{\max}\left(Q^{\left(K\right)}\right)$ denotes the unit eigenvector corresponding to the maximum eigenvalue of the weighted covariance matrix $Q^{\left(K\right)}$.
3. Weight Update Based on Huber M-estimator. The weights are iteratively updated using the Huber M-estimator to reduce the influence of contour centroids with large fitting residuals [42]. The residuals are defined as the orthogonal distances from the contour centroids to the current estimated central axis, which are subsequently used to determine the robustness weights. According to the magnitude of the residuals, the Huber function assigns full weights to points with small deviations and gradually decreases the weights of points with large deviations, as expressed in Equation (21).
  (21) $$w_{i}^{\left(k+1\right)}=\left\{\begin{array}{c} 1,\;\;\;d_{i}^{\left(k\right)}\leq \delta_{h}\\ \frac{\delta_{h}}{d_{i}^{\left(k\right)}},\;\;d_{i}^{\left(k\right)}>\delta_{h} \end{array}\right.$$
  where $w_{i}^{\left(k+1\right)}$ denotes the updated weight assigned to the i−th contour centroid for the next iteration, $d_{i}^{\left(k\right)}$ represents the orthogonal distance from the i−th centroid to the current axis and $\delta_{h}$ is the Huber threshold.
4. Convergence Criterion of the Iteration. When the algorithm converges, the iteration is terminated, and the unit direction vector of the central axis is output. Otherwise, the iteration index is updated, and the process returns to Equation (10). The convergence criterion is defined in Equation (22).
  (22) $$\left\|d^{\left(k\right)}-d^{\left(k-1\right)}\right\|\leq \partial \;\mathrm{or}\;k\geq k_{\max}$$
  where ∂ denotes the convergence threshold for the variation in the unit direction vector of the central axis between two consecutive iterations; and $k_{\max}$ represents the preset maximum number of iterations.

#### 2.3.3. Computation of Inclination Parameters of the Lattice Steel Tower Structures Central Axis

1. Calculation of the Inclination Azimuth of the Central Axis. In the station-centered spatial coordinate system (O-XYZ), the unit vector of the x-axis is denoted as $X=\left[1,0,0\right]$. The inclination azimuth angle β is the coordinate azimuth. It is defined as the horizontal angle formed by a line, whose inclination angle is located in the planar rectangular coordinate system based on the target object’s position, measured clockwise from this line to the coordinate vertical axis (north direction). Equivalently, it can also be defined as the horizontal angle between the central axis unit vector $d^{\left(k\right)}$ and X, which can be obtained through vector operations, as expressed in Equation (23).
  (23) $$\beta =\mathrm{arc}\cos\left(\frac{X\cdot d^{\left(k\right)}}{\left|X\right|\cdot \left|d^{\left(k\right)}\right|}\right)$$
2. Calculation of the Inclination Angle of the Central Axis. In the station-centered spatial coordinate system (O-XYZ), the unit vector of the z-axis is denoted as $Z=\left[0,0,1\right]$. The inclination angle φ of the central axis is defined as the vertical angle between the central axis unit vector $d^{\left(k\right)}$ and Z, which can be obtained through vector operations, as expressed in Equation (24).
  (24) $$\varphi =\mathrm{arc}\cos\left(\frac{Z\cdot d^{\left(k\right)}}{\left|Z\right|\cdot \left|d^{\left(k\right)}\right|}\right)$$
3. Calculation of the Inclination Degree of the Central Axis. According to the definition of inclination degree, the inclination value corresponding to the central axis can be obtained from Equation (25).
  (25) I=tan(φ)
4. Stability and consistency evaluation. To quantitatively evaluate the consistency of inclination values under different cross-sectional slicing schemes, the mean value and mean deviation were calculated for each scheme. Assuming that V cross-sectional slicing schemes are considered in total, the mean inclination value is defined as Equation (26).
  (26) $$\bar{I}=\frac{1}{V}\sum_{u=1}^{V}I_{u}$$
  where V denotes the total number of cross-sectional slicing schemes considered in the comparative evaluation, $I_{u}$ denotes the inclination value obtained under the u−th scheme, and $\bar{I}$ represents the mean value of the results from all schemes.
5. Furthermore, the mean deviation is employed to evaluate the dispersion of the results, as shown in Equation (27).
  (27) $$MD=\frac{1}{V}\sum_{u=1}^{V}\left|I_{u}-\bar{I}\right|$$
  where MD denotes the mean deviation. A smaller MD value indicates a lower degree of dispersion among the results obtained under different cross-sectional slicing schemes, implying better consistency and robustness of the proposed algorithm.

## 3. Experiment

### 3.1. Experimental Design

**Figure 4:**
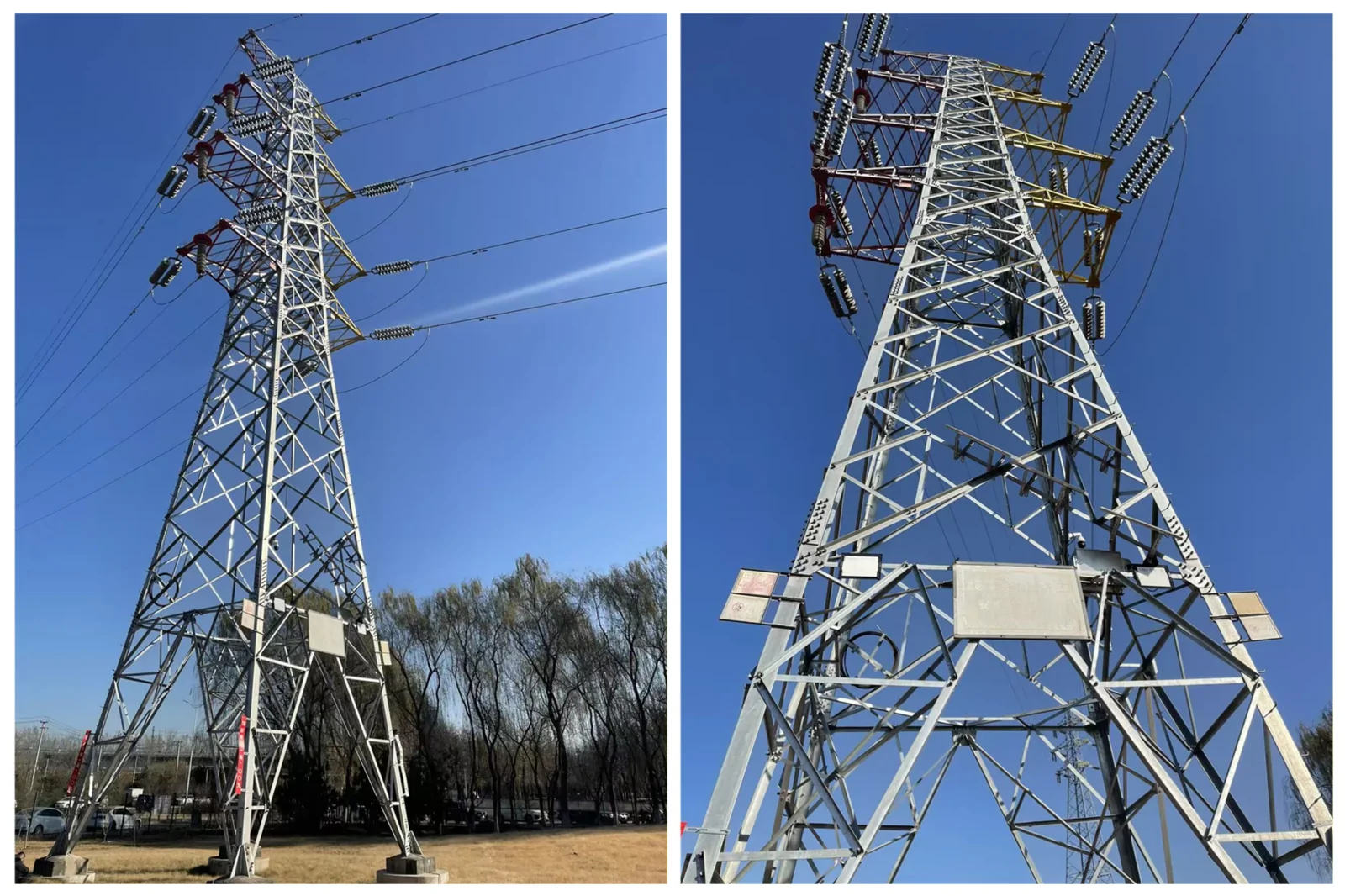
Experimental object.

*A*n in-service lattice steel tower located along a 110 kV overhead transmission line in Qingyuan Park was selected as the experimental object in this study. The tower is a gantry-type angle tower with a total height of approximately 30 m and adopts a four-legged open-frame lattice structure, consisting mainly of main members, diagonal bracing members, and horizontal connecting members. The tower body comprises various structural members and exhibits pronounced open-frame characteristics, providing a representative experimental scenario for LiDAR point cloud-based cross-sectional geometric feature extraction and inclination detection. Figure 4 shows the lattice steel tower selected as the experimental object in this study.

**Figure 5:**
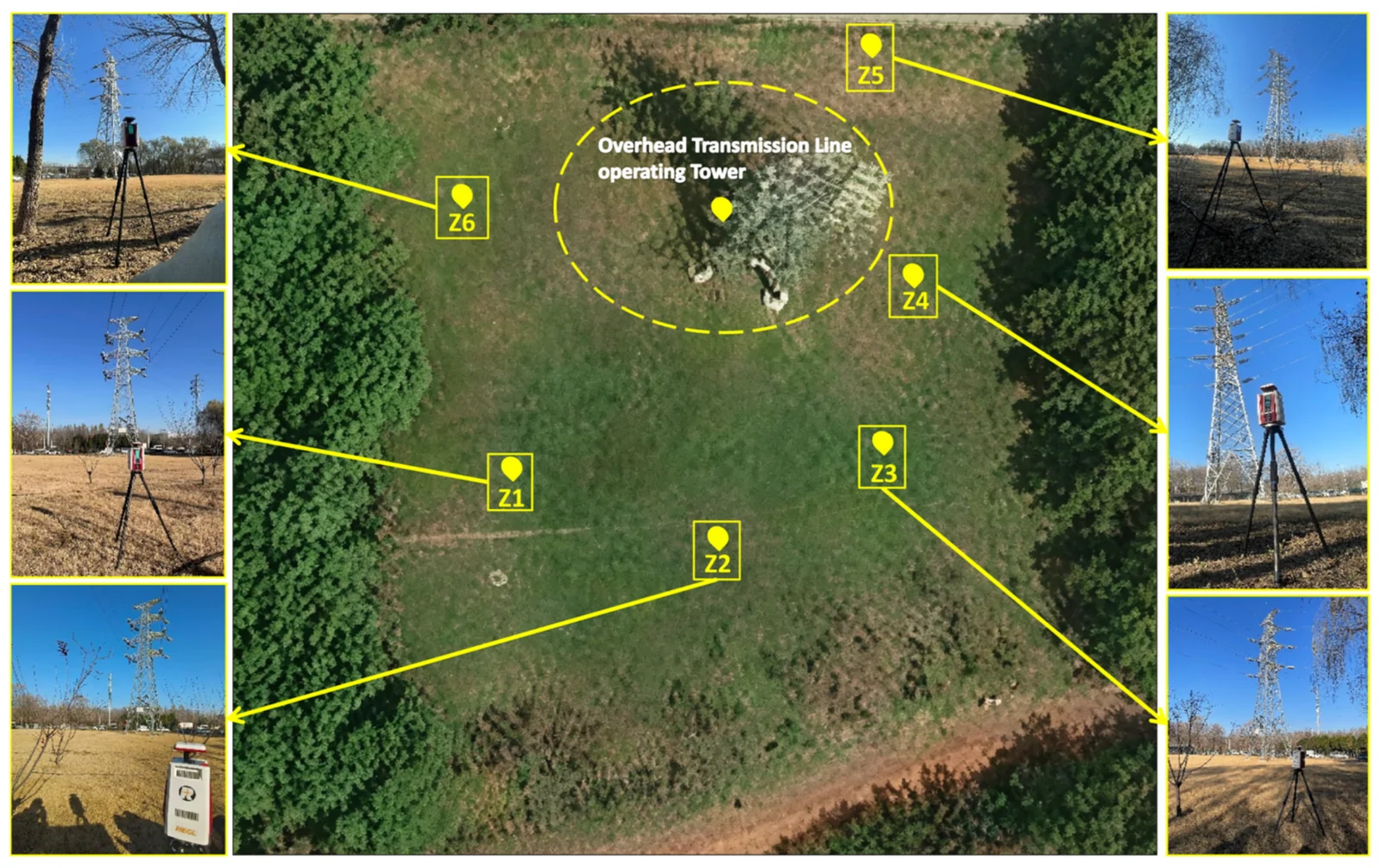
Schematic diagram of the scanning station layout at the experimental site.

To investigate the performance differences in different cross-sectional feature extraction algorithms for inclination detection of lattice steel towers, MATLAB R2024b was used to develop separate data-processing modules for the RANSAC and Marching Square algorithms. Different cross-sectional slicing schemes were designed to conduct a systematic comparative analysis of the two algorithms. Point cloud data of an in-service tower along an overhead transmission line in Qingyuan Park were acquired using a terrestrial laser scanner, and the schematic diagram of the scanning-station locations is shown in Figure 5. A Rigel VZ-600i scanner (manufactured by RIEGL LMS GmbH, Horn, Austria), with a measurement range of 0.5–1000 m, three-dimensional positional accuracy of 3 mm at 50 m and 5 mm at 100 m, a field of view of 360° × 105°, a vertical angular resolution of <0.0007°, a horizontal angular resolution of <0.0005°, three-dimensional coordinate measurement accuracy of (5.1, 5.1, 1.2) mm at 100 m, a data acquisition rate of 2.2 million points/s, and a scanning capacity of up to 60 stations per hour. Six scanning stations were established at suitable locations 50–100 m from the in-service transmission tower with clear visibility of the tower body (The camera exposure time was set to 1/10 s, the measurement rate was set to 1200 kHz, and the scanning mode was set to Panorama_40, in which a laser beam was emitted at an angular interval of 0.04°, corresponding to a linear resolution of 0.04° and a coarse-scan resolution of 0.04°), and full-range scans were performed to acquire the raw point cloud data of the tower. Z1–Z6 in Figure 5 denote the six scanning-station locations.

### 3.2. Preprocessing of LiDAR Point Cloud Data

**Figure 6:**
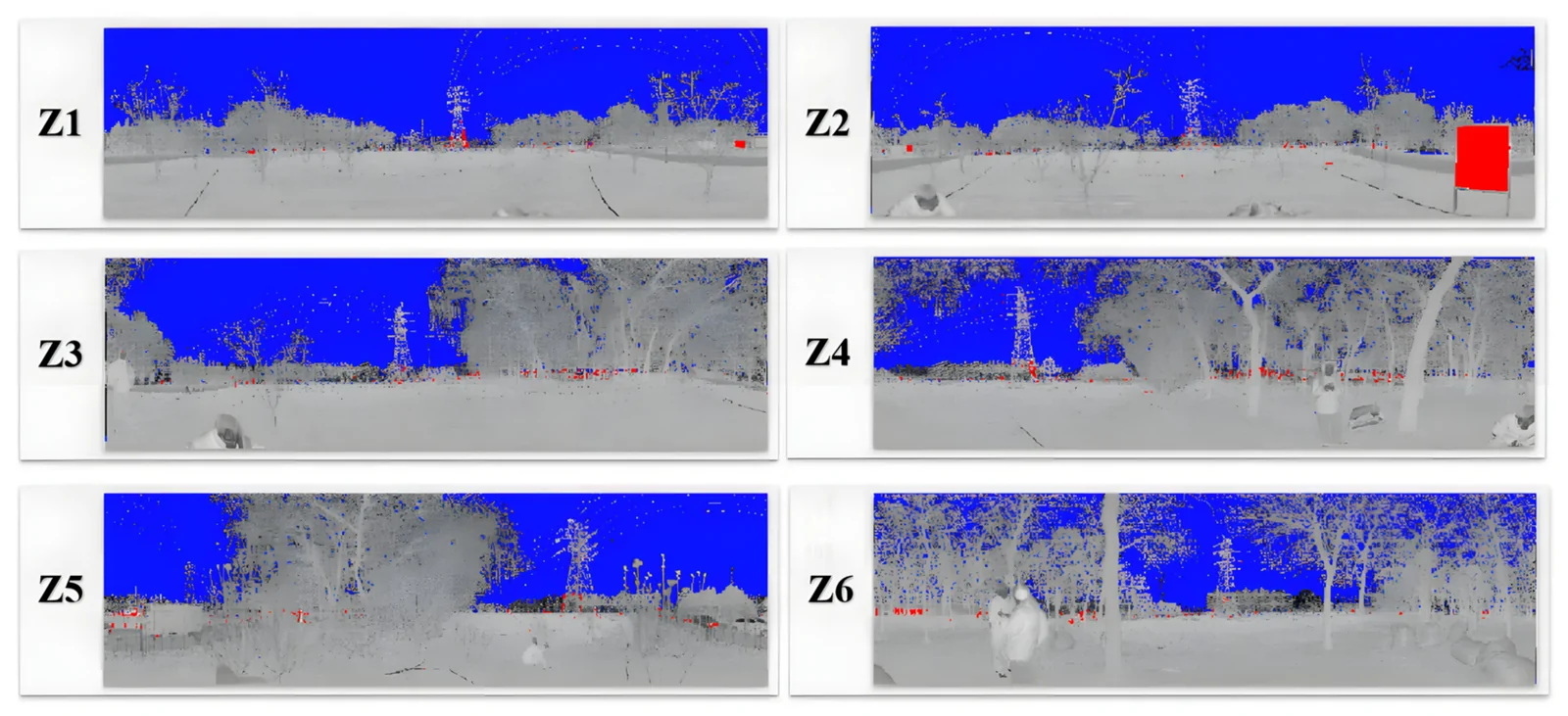
Raw laser point cloud data.

LiDAR point cloud data of the overhead transmission line operating tower were acquired from six scanning stations. The original LiDAR point cloud data acquired from each scanning station are shown in Figure 6.

**Figure 7:**
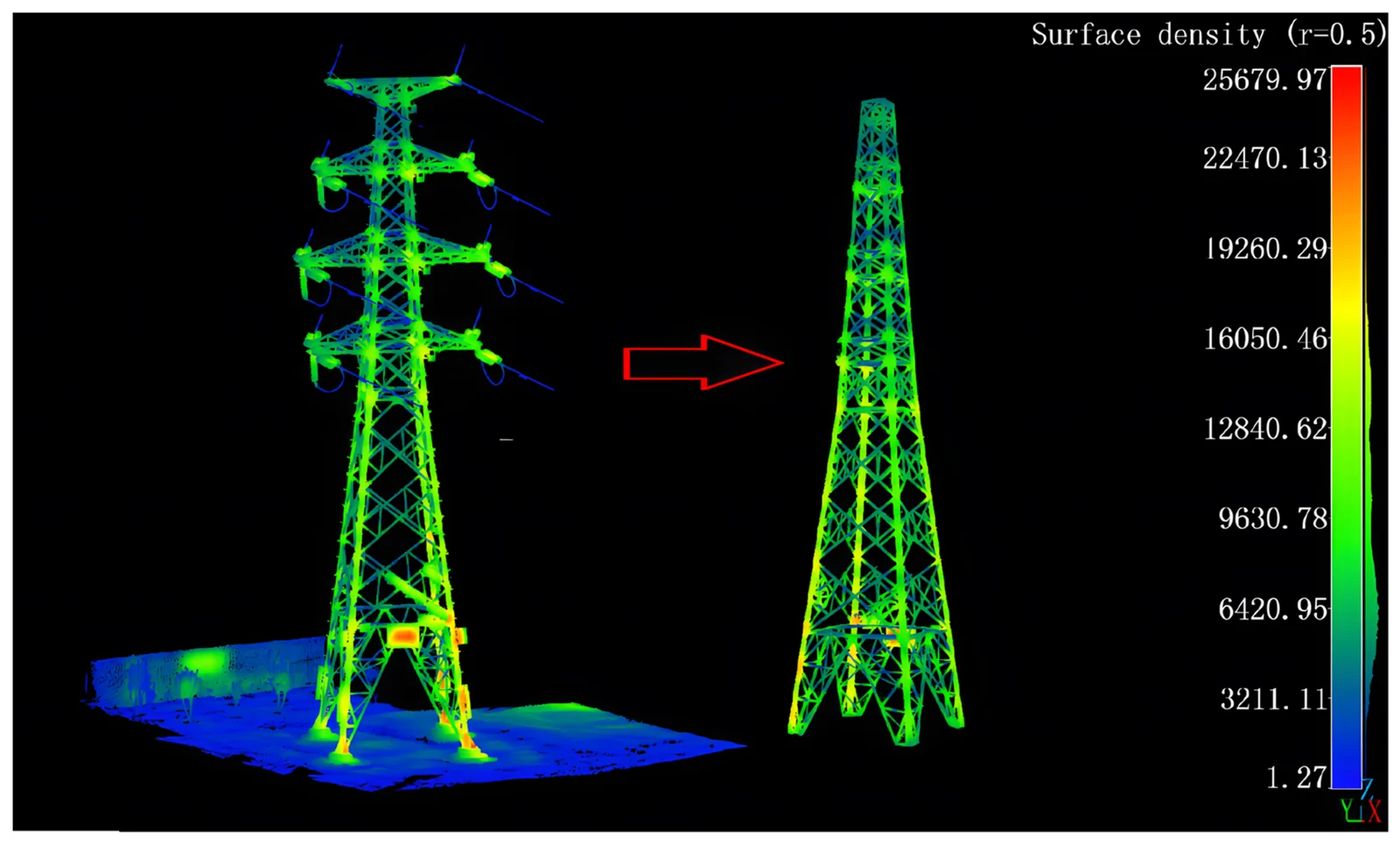
Effective point cloud data of tower body.

The original point cloud data acquired from the six scanning stations shown in Figure 5 were registered using the accompanying RiSCAN PRO 2.19.1 software and then exported in LAS format. The first scanning station was used as the reference station. The other five scanning stations were individually registered to the first scanning station. Four groups of feature points that were non-coplanar in three-dimensional space and differed in both elevation and horizontal position were selected in RiSCAN PRO 2.19.1 for initial registration. The initial registration results were then further optimized through multi-station adjustment in RiSCAN PRO 2.19.1. All registered point clouds were ultimately transformed into the station-centered spatial coordinate system (O-XYZ). The effective point cloud data of the in-service transmission tower are shown in Figure 7.

As shown in Figure 7, the point cloud data of the overhead transmission line operating tower before preprocessing still contained environmental interference, including cross-arms and surrounding vegetation. To improve the accuracy of subsequent inclination detection, the high-precision point cloud data in LAS format were further processed using CloudCompare v2.13.2 for point cloud segmentation, thereby extracting the valid point cloud data of the tower body.

### 3.3. Design of Cross-Sectional Slicing Schemes

*T*o investigate the influence of cross-sectional sampling strategies on inclination detection results and to evaluate the robustness of the feature extraction methods based on the RANSAC and Marching Square algorithms under different sampling conditions, four cross-sectional slicing schemes were designed using CloudCompare v2.13.2. According to Equation (2), the slice thickness was uniformly set to 0.05 m for all schemes. For each scheme, the RANSAC and Marching Square algorithms were separately employed to extract the corresponding cross-sectional geometric features, from which the centroid coordinates, central axis, and inclination values were calculated. The robustness of each algorithm was evaluated by comparing the inclination values obtained under the four slicing schemes. Specifically, the mean inclination and mean deviation (MD) were calculated to quantify the overall consistency and dispersion of the results, respectively. A smaller mean deviation indicates that the estimated inclination is less affected by variations in the cross-sectional sampling conditions, suggesting that the corresponding feature extraction method exhibits greater robustness. The detailed configurations of the four slicing schemes are summarized in Table 1.

The rationale behind the selection of the four slicing schemes is described as follows. Scheme A adopts a 1 m interval to represent a high-density sampling strategy, where abundant cross-sectional information is available for accurate axis reconstruction. Scheme B uses a 2 m interval as a moderate-density sampling strategy, aiming to balance detection accuracy and computational efficiency. Scheme C selects transverse bracing sections because these locations correspond to important structural nodes where steel members intersect and geometric variations are relatively significant, making them representative structural features for tower monitoring. Scheme D adopts a 4 m interval to simulate sparse sampling conditions and evaluate the robustness of the algorithms when fewer cross-sectional observations are available.

## 4. Results

### 4.1. Cross-Sectional Contour Extraction Results

**Figure 8:**
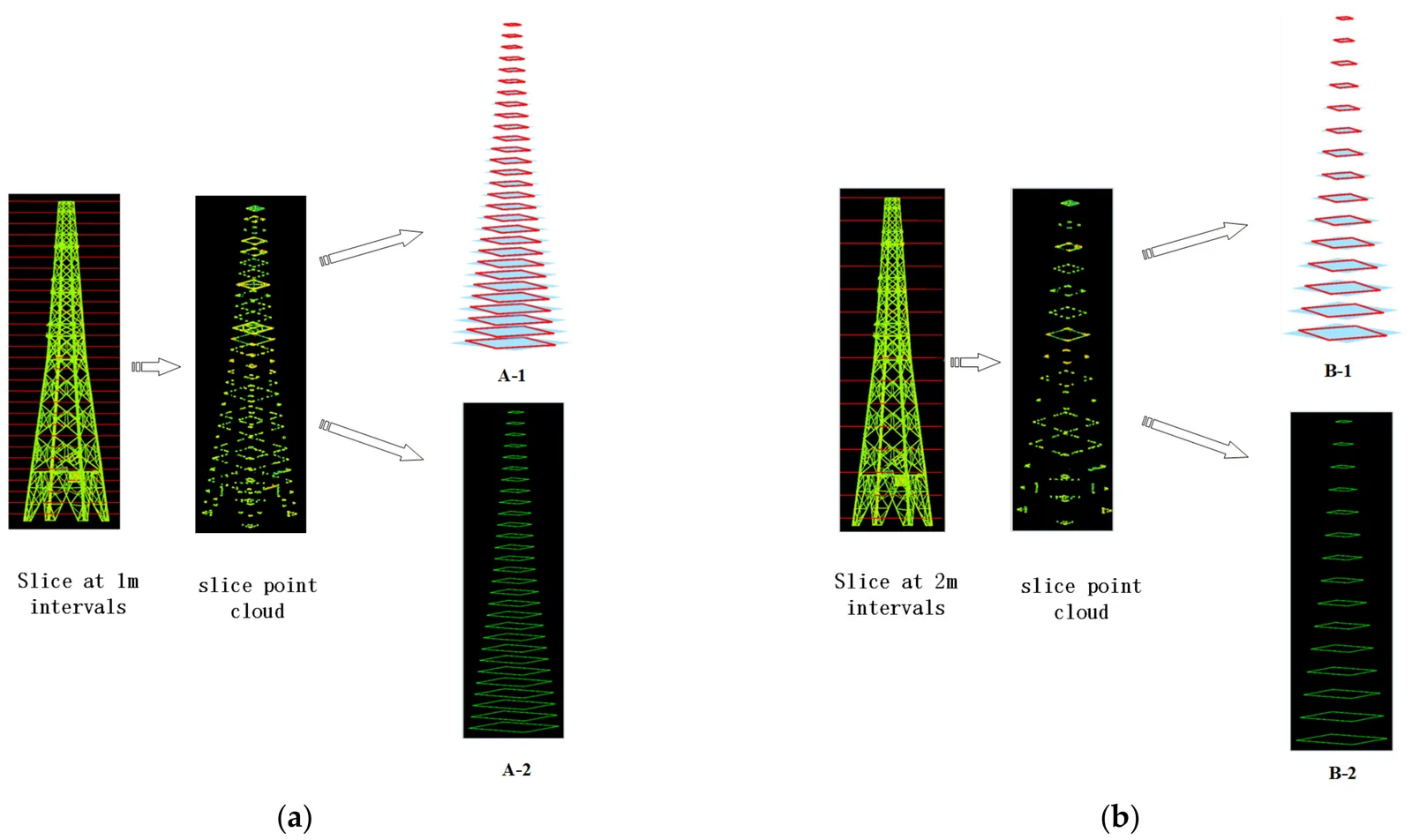

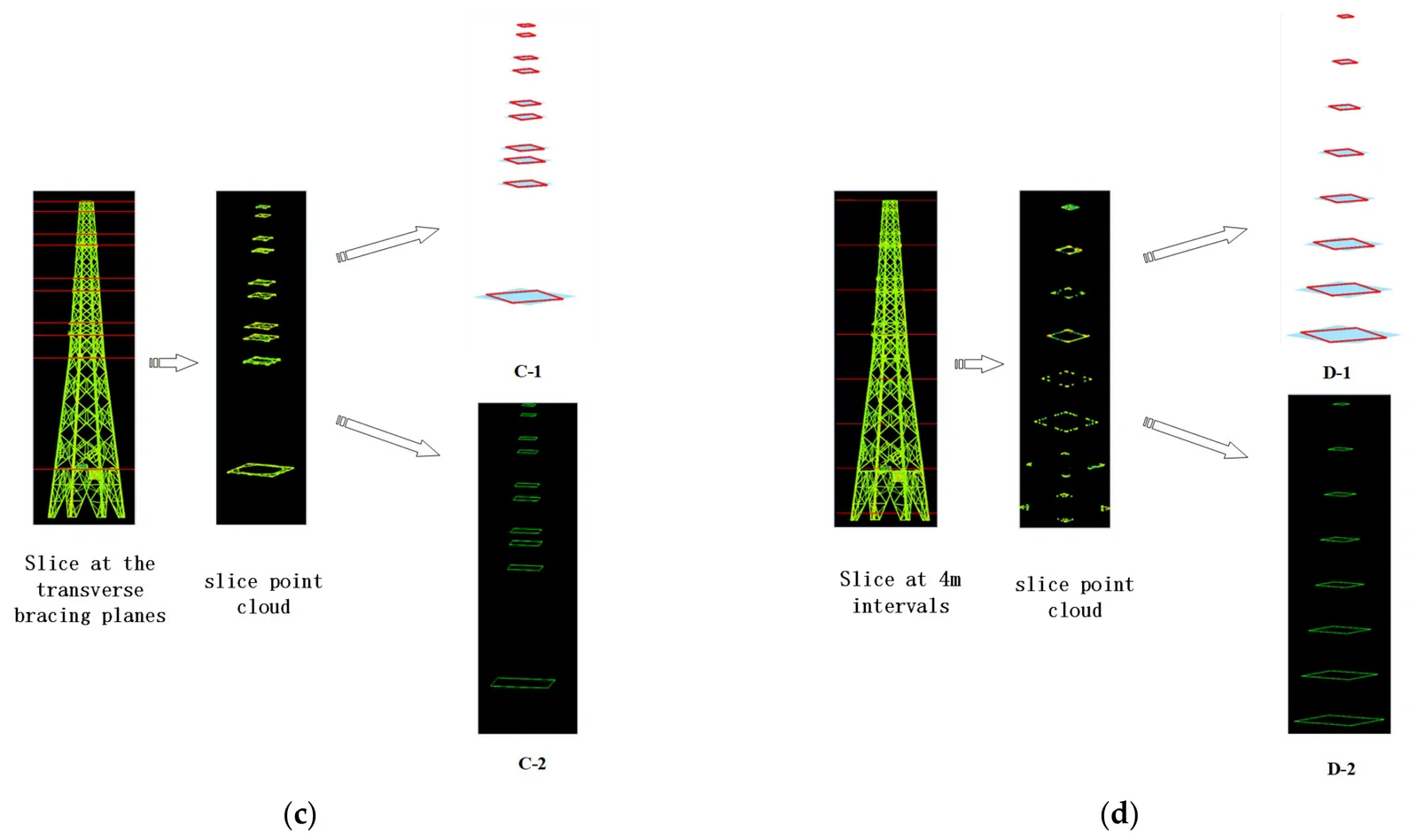
Four slicing segmentation schemes and extraction results. (**a**) Results corresponding to Scheme A; (**b**) results corresponding to Scheme B; (**c**) results corresponding to Scheme C; (**d**) results corresponding to Scheme D.

*A*ccording to the four designed cross-sectional slicing schemes, the cross-sectional contours were extracted using the RANSAC and Marching Square algorithms, respectively. The corresponding contour extraction results are presented in Figure 8.

### 4.2. Inclination Results

**Figure 9:**
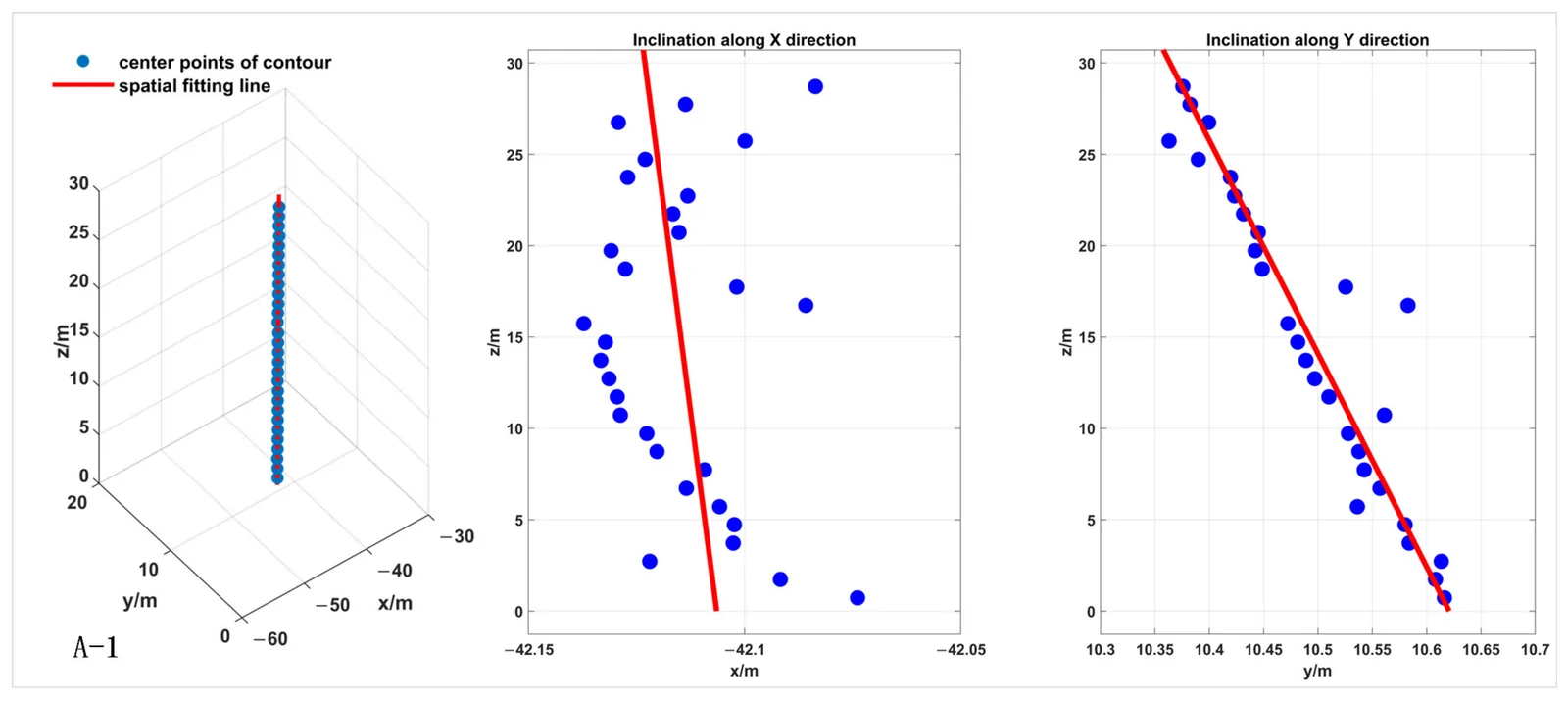

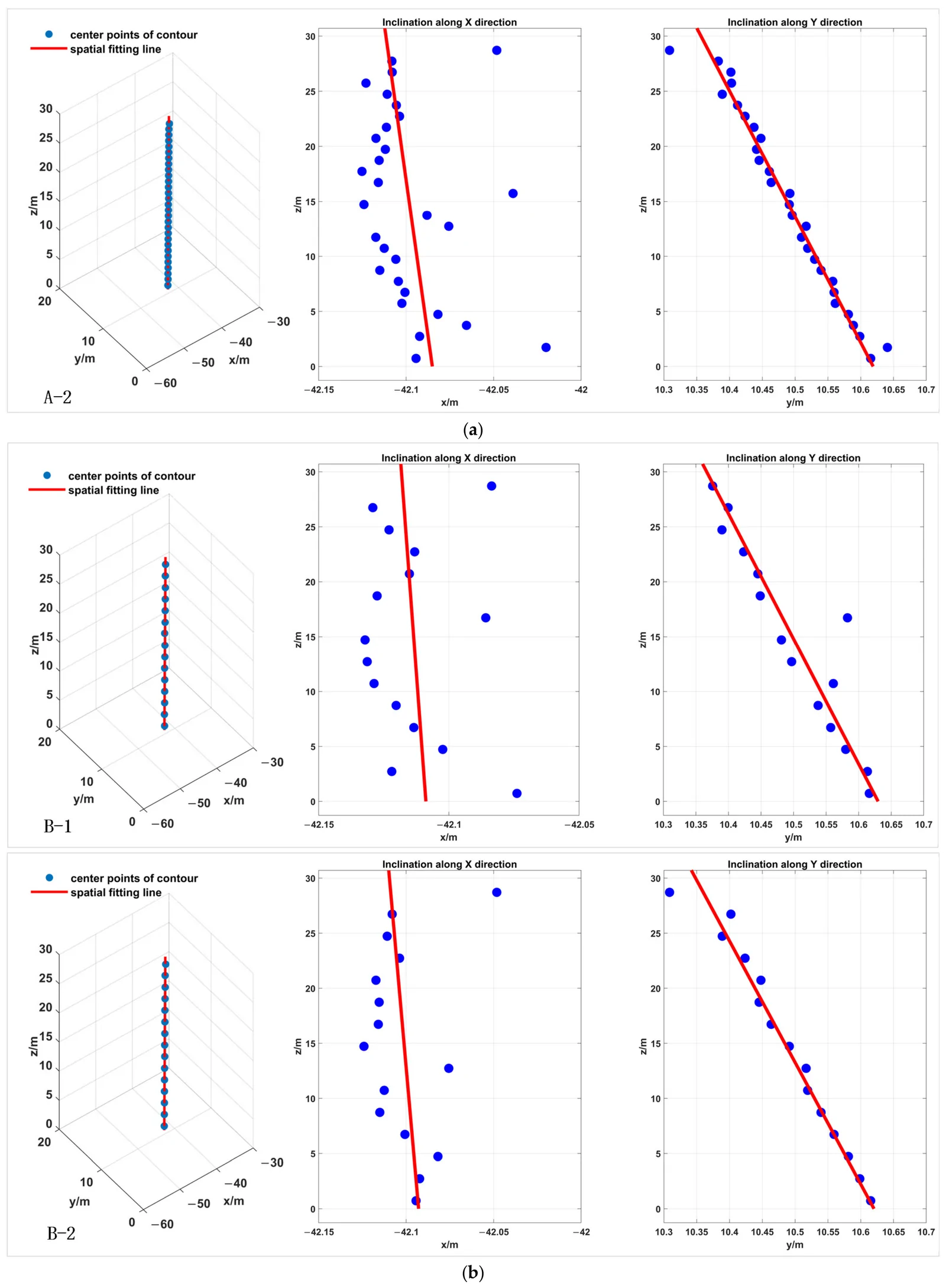

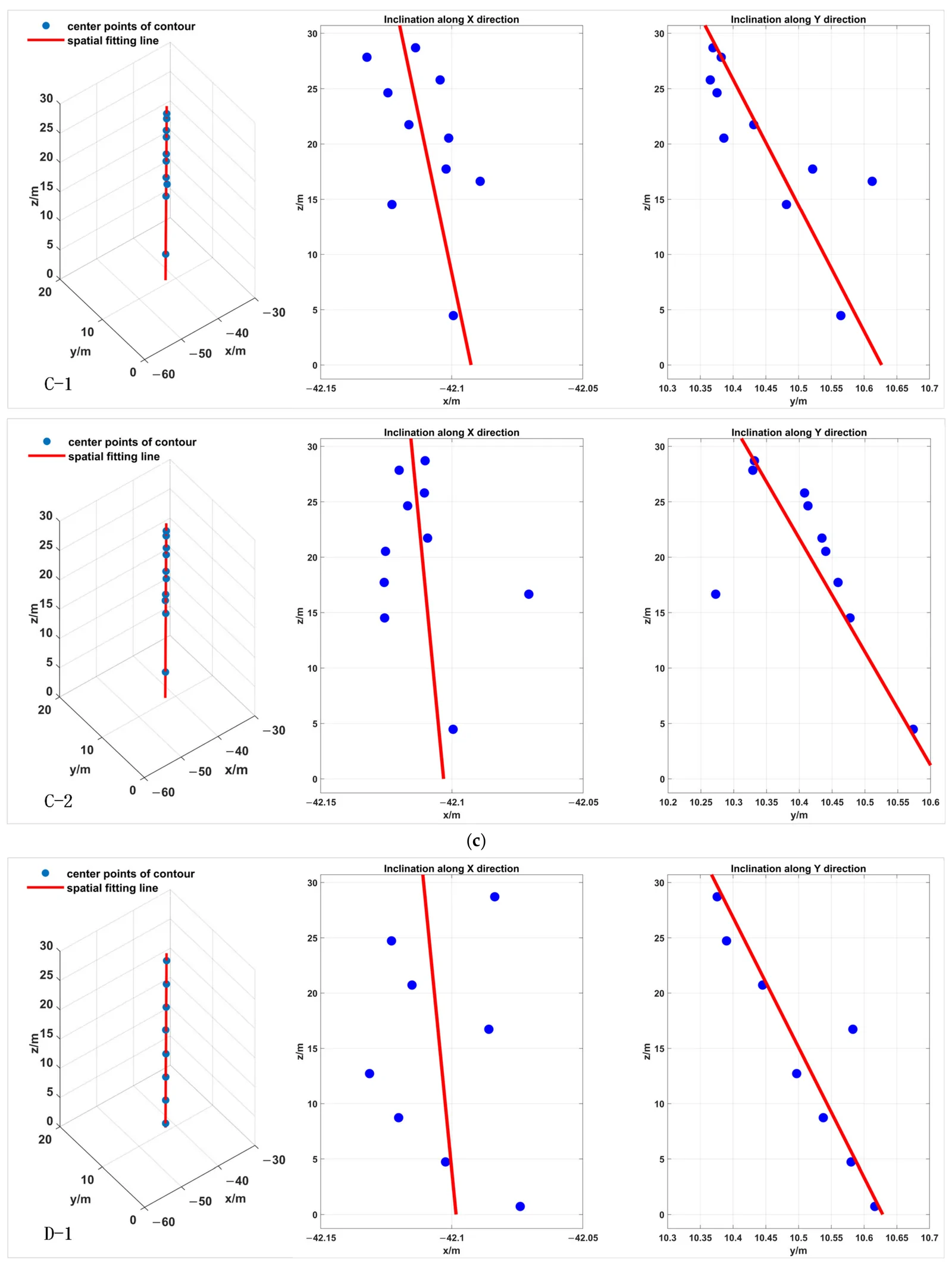

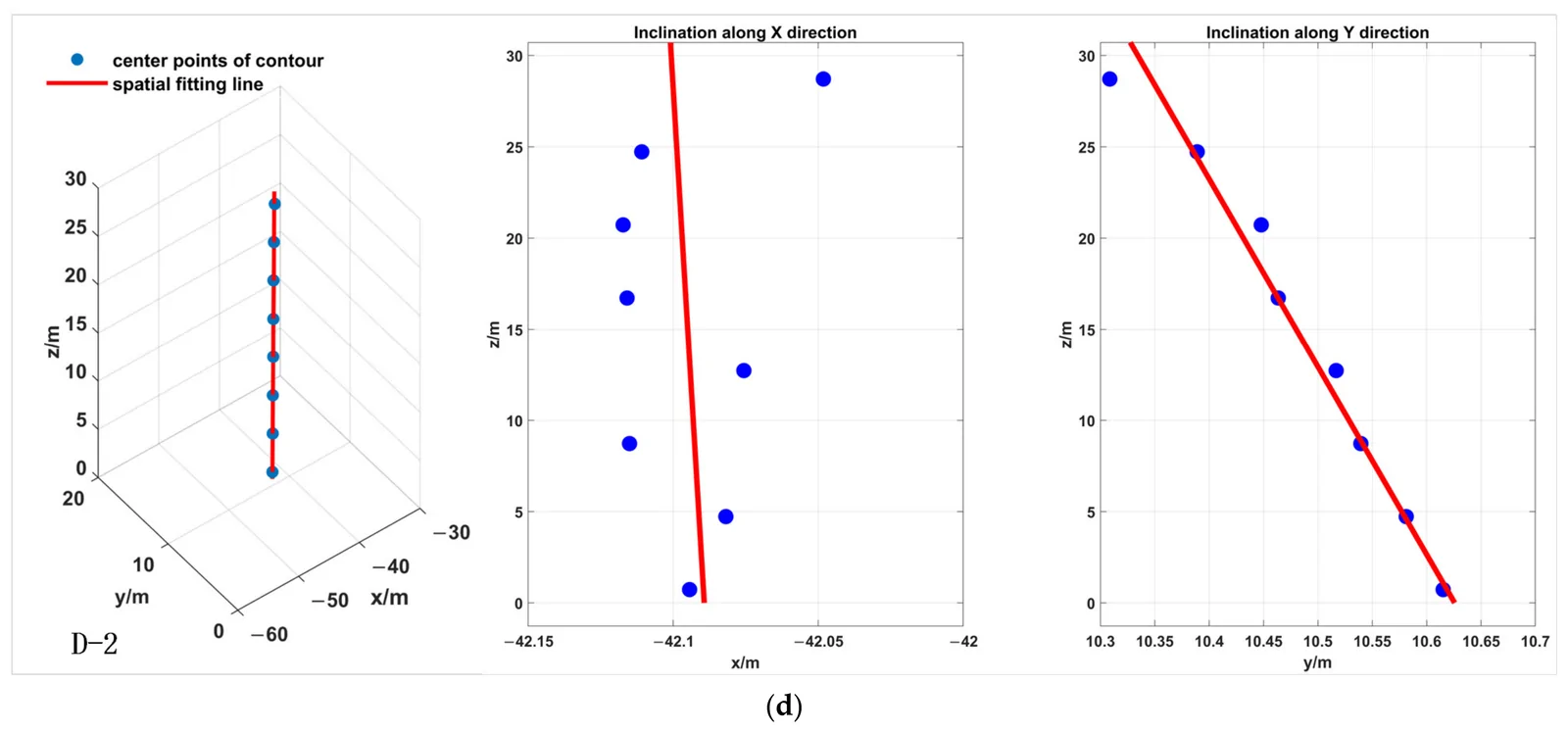
Inclination Results. (**a**) Results corresponding to Scheme A; (**b**) results corresponding to Scheme B; (**c**) results corresponding to Scheme C; (**d**) results corresponding to Scheme D.

*F*or Schemes A–D, the RANSAC and Marching Square algorithms were respectively employed to perform spatial line fitting on the extracted cross-sectional centroids, thereby obtaining the corresponding central axes of the tower body. The fitting results are shown in Figure 9. The figure provides an intuitive comparison of the fitting stability of the two methods under different sampling densities.

The spatial lines of the central axis corresponding to the four schemes shown in Figure 8 were used to determine their direction vectors. Vector operations with the x- and *z*-axis in the station-centered coordinate system were then performed to derive the inclination azimuth, inclination angle, and inclination magnitude. The corresponding inclination values for the four schemes are presented in Table 2.

As shown in Table 1, the tower inclination values obtained using the RANSAC algorithm under the four cross-sectional slicing schemes were 8.58‰, 8.78‰, 8.82‰, and 8.52‰, respectively, with an average value of 8.68‰ and a range of 0.30‰. Under different slicing schemes, the inclination results derived from the RANSAC algorithm exhibited relatively smooth variations, demonstrating good consistency. In contrast, the inclination values obtained using the Marching Square algorithm were 8.79‰, 9.08‰, 9.79‰, and 9.72‰, respectively, with an average value of 9.35‰ and a range of 1.00‰. The inclination results obtained by both algorithms under the four slicing schemes satisfied the requirement specified in DL/T 741–2019 (Code of Practice for Operation of Overhead Transmission Lines) [43], which limits the tower inclination to no more than 10‰. These results indicate that both inclination detection methods based on cross-sectional feature extraction are capable of meeting the requirements for practical engineering inclination assessment.

## 5. Discussion

**Figure 10:**
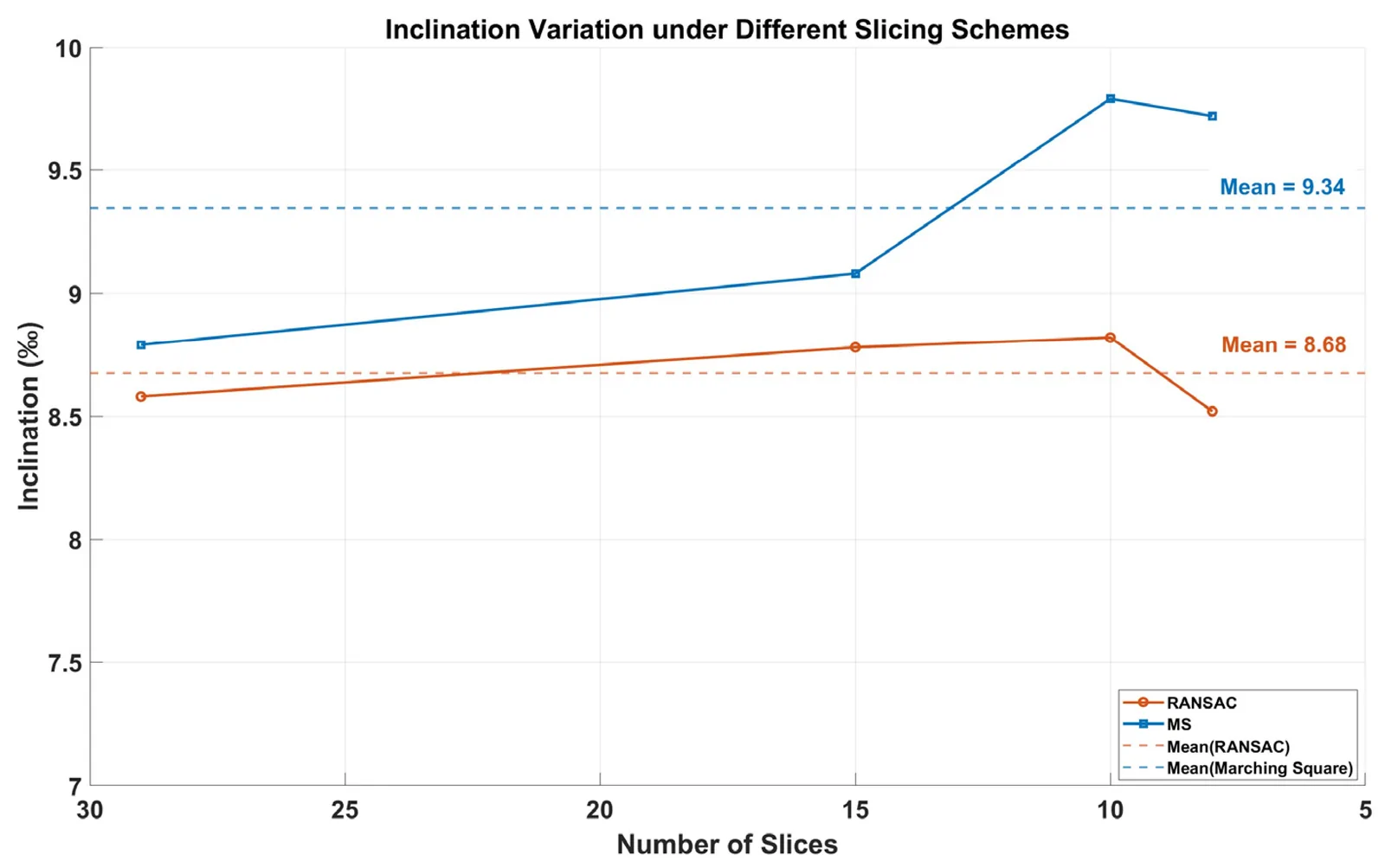
**C**omparative analysis of inclination values.

To evaluate the detection stability of the RANSAC and Marching Square algorithms under different cross-sectional slicing strategies, both algorithms were applied to four cross-sectional sampling schemes with 29, 15, 10, and 8 slices, respectively, to calculate the tower inclination. The inclination results obtained under different schemes were compared and analyzed, as shown in Figure 10.

Based on the inclination results obtained from different schemes and the mean inclination deviations under various cross-sectional slicing strategies, the following observations can be drawn:

1. Both algorithms were capable of achieving tower inclination estimation under different cross-sectional slicing schemes, and the overall variation trends of the obtained inclination values were consistent. As the number of slices decreased, the inclination results obtained by both algorithms exhibited certain fluctuations. Among them, the RANSAC algorithm showed relatively smoother variations and a smaller range of inclination values, indicating better result consistency. The Marching Square algorithm effectively utilized the boundary information of cross-sectional point clouds for contour feature extraction; however, its contour reconstruction process relies on the continuity of point cloud distribution, resulting in relatively larger fluctuations under different slicing schemes. These differences reflect the distinct feature extraction characteristics of the two algorithms under complex point cloud conditions.
2. The differences between the mean inclination values obtained by the two algorithms varied among the four slicing schemes, with absolute differences of 0.21‰, 1.20‰, 1.17‰, and 1.20‰ for Schemes A, B, C, and D, respectively. Scheme A exhibited the smallest difference, while the differences observed in Schemes B–D were relatively similar. These results indicate that the influence of cross-sectional sampling on inclination estimation is not simply proportional to the number of sampled sections. When fewer cross-sectional observations are available, individual feature extraction errors may have a greater influence on central axis reconstruction; however, the magnitude of this influence also depends on the spatial distribution and geometric representativeness of the selected sections. Therefore, the cross-sectional sampling strategy should be determined according to the structural geometry and point cloud quality rather than solely according to the number of slices. In practical applications, cross-sectional locations should preferentially cover structural joints, bracing connections, geometric transition regions, and the upper and lower portions of the tower, while excessive sampling in geometrically homogeneous regions can be reduced to balance detection reliability and computational efficiency.
3. The mean deviations of the inclination results obtained by the two algorithms under the four slicing schemes were 0.12‰ for RANSAC and 0.41‰ for Marching Square, respectively. Under the experimental conditions of this study, the RANSAC algorithm exhibited lower result dispersion under different slicing configurations, indicating that its feature extraction mechanism based on inlier–outlier consistency evaluation can maintain relatively stable performance in the presence of outliers and local data gaps. In contrast, the Marching Square algorithm extracts cross-sectional boundaries by reconstructing spatial contour information and can better preserve the geometric shape of cross-sections when the point cloud distribution is complete and the boundaries are continuous. However, it is more sensitive to local data missing and boundary anomalies. Therefore, the two algorithms exhibit different advantages in terms of feature extraction strategies.
4. Overall, under the experimental conditions of this study, both algorithms yielded tower inclination detection results that satisfied the relevant engineering criterion, although they exhibited different characteristics in terms of result stability and cross-sectional geometric representation. According to DL/T 741—2019 (Code of Practice for Operation of Overhead Transmission Lines), the allowable inclination limit for the angle-steel lattice tower considered in this study is 10‰. The investigated tower is an approximately 30 m high, 110 kV angle-steel lattice tower. The inclination values obtained using the RANSAC and Marching Square algorithms under all four cross-sectional sampling schemes were below this limit, indicating that both methods can satisfy the engineering requirements for inclination-state assessment of the investigated tower under the experimental conditions. The 10‰ criterion represents the allowable structural inclination limit rather than the measurement accuracy requirement of the point cloud measurement system itself. While both methods satisfied the engineering criterion, they exhibited distinct applicability characteristics. The RANSAC algorithm showed a smaller variation in inclination values across the four cross-sectional sampling schemes, indicating better consistency of the detection results. This suggests that its feature extraction mechanism, based on inlier selection within a fitted plane model, is relatively robust to local point cloud incompleteness and outliers. In contrast, the Marching Square algorithm can represent the geometric shape of cross-sectional boundaries more intuitively through continuous isocontours, but its results are more sensitive to the spatial distribution of the point cloud and the completeness of local boundaries, resulting in relatively larger variations across different sampling schemes. Therefore, under the same engineering criterion, algorithm selection should also consider point cloud quality, structural geometric characteristics, and the stability of the detection results.

## 6. Conclusions

This study focused on the LiDAR point cloud-based inclination detection of lattice steel towers and established an experimental framework for evaluating the performance of cross-sectional feature extraction algorithms. The applicability of the RANSAC and Marching Square algorithms for lattice tower inclination detection was comparatively analyzed. The main conclusions are summarized as follows:

1. A point cloud-based inclination detection framework for lattice steel towers was established based on cross-sectional feature extraction, enabling a complete processing workflow from cross-sectional feature extraction to structural axis reconstruction and inclination parameter estimation. Through point cloud registration, redundancy removal, and noise filtering, combined with multiple cross-sectional slicing strategies, the RANSAC and Marching Square algorithms were respectively employed to extract cross-sectional geometric features. Subsequently, the spatial distribution of the extracted centroid points was fitted using the IRLS algorithm to determine the tower central axis direction vector and three-dimensional inclination parameters. This framework provides a unified experimental basis for investigating the influence of different feature extraction algorithms on inclination detection results.
2. Field experimental results demonstrated that both the RANSAC and Marching Squares algorithms were capable of detecting the inclination of the investigated 110 kV angle-steel lattice tower. The inclination values obtained under all four cross-sectional sampling schemes were below the allowable inclination limit of 10‰ specified in DL/T 741—2019 (Code of Practice for Operation of Overhead Transmission Lines), indicating that both methods were capable of providing an engineering basis for inclination-state assessment under the experimental conditions of this study.
3. The two algorithms exhibited different levels of stability in inclination estimation. The range and mean deviation of the inclination results obtained by the RANSAC algorithm were 0.30‰ and 0.12‰, respectively, whereas those obtained by the Marching Square algorithm were 1.00‰ and 0.41‰, respectively. Under the experimental conditions investigated in this study, the RANSAC algorithm, relying on an inlier–outlier consistency evaluation mechanism, showed lower result dispersion and better consistency among different slicing schemes. This behavior indicates that RANSAC can effectively reduce the influence of outliers and local data gaps during cross-sectional feature extraction for the tested lattice steel tower. In contrast, the Marching Square algorithm extracts boundary features based on spatial contour continuity and exhibits stronger geometric representation capability when the point cloud distribution is complete; however, its performance may be affected by local boundary variations. Therefore, the two algorithms exhibit different characteristics in cross-sectional feature extraction, and their relative advantages may depend on the structural configuration and point cloud quality.
4. Although the experimental object in this study was an angle-type overhead transmission tower, the proposed algorithm comparison framework provides a potential approach for evaluating cross-sectional feature extraction methods of lattice steel structures. For transmission towers, communication towers, and other open-frame steel structures with identifiable cross-sectional geometric features, the proposed framework may provide a reference for algorithm selection, provided that sufficient point cloud quality and structural feature information are available. However, the current conclusions are derived from a single field experiment, and further validation using different tower types, measurement environments, and point cloud conditions is required before broader applicability can be confirmed.

Future research will further advance the proposed framework in terms of structural-type expansion and refined characterization of deformation features. The present study considers only one in-service overhead transmission line tower as the experimental object. Future work will extend the investigation to typical tall lattice steel structures, including communication towers, radio and television towers, tower-type building structures, and observation towers, with field experiments and comparative analyses under diverse structural conditions to further verify the applicability and stability of different feature extraction algorithms and enhance the generalizability of the conclusions. The current method mainly fits a spatial straight line to the cross-sectional centroids at different elevations using IRLS to characterize the overall inclination of the tower, while its ability to describe local bending, torsion, and nonlinear deformation remains limited. Future work will construct a spatial polyline centerline based on the extracted cross-sectional centroids and combine the center positions, boundary orientations, and cross-sectional attitudes at different elevations to extend the assessment from overall inclination to local deformation characterization. The local inclination angle will also be utilized for depth assessment. Cross-sectional locations without transverse arms or other additional structural components will be preferentially selected to minimize structural interference and improve the reliability of feature extraction. While the present study focuses on the performance comparison between RANSAC and Marching Square for inclination monitoring of tall slender structures, future work will also explore other promising approaches for cross-section extraction. For example, a geometrically simpler procedure can be considered: First, define the vertical direction and create a horizontal slice at the required height. The points within this slice can then be projected onto a horizontal plane, rather than onto a regression plane. The shape and centre of the cross-section can subsequently be estimated directly in this horizontal plane. This approach may be more robust when dealing with incomplete point clouds, as it avoids the potential inaccuracies introduced by fitting a regression plane that may not represent a true horizontal section. We plan to implement this horizontal-slice projection method in future studies and systematically compare its performance with that of the RANSAC-based and Marching Square-based methods in terms of accuracy, stability, and computational efficiency. Such comparative analyses will provide valuable insights into the effectiveness and applicability of each method, and help to identify their respective strengths and limitations in verticality monitoring. Systematic field experiments under different structural types, deformation levels, scanning distances, and point-cloud quality conditions will also be conducted, together with cross-validation using multi-source measurement techniques, to further evaluate the stability, reliability, and applicability of the proposed method.

## Figures and Tables

**Table 1:** Configuration of different cross-sectional slicing schemes.

Scheme	Slicing Strategy	Slicing Interval	Number of Slices	Results from RANSAC and Marching Squares
A	Horizontal slicing along *z*-axis	1 m interval	29	A-1/A-2
B	Horizontal slicing along *z*-axis	2 m interval	15	B-1/B-2
C	Slicing at transverse bracing planes	Structural bracing positions	10	C-1/C-2
D	Horizontal slicing along *z*-axis	4 m interval	8	D-1/D-2

In each scheme, the suffixes “−1” and “−2” represent the inclination results obtained using the RANSAC and Marching Squares algorithms, respectively.

**Table 2:** Tower body inclination detection parameter results for the two algorithms.

Scheme		Scheme A	Scheme B	Scheme C	Scheme D
Indicator					
Number of cross-sectional slices		29	15	10	8
Number of slice contours		29	15	10	8
Number of contour centroid points		29	15	10	8
Unit Direction Vector of the Tower Central Axis	1 (RANSAC)	−0.0005531, −0.0085634, 0.9999632	−0.0005531, −0.0085634, 0.9999632	−0.0008879, −0.0087770, 0.9999611	−0.0004132, −0.0085101, 0.9999637
	2 (Marching Square)	−0.0008841, −0.0087468, 0.9999614	−0.0005556, −0.0090657, 0.9999588	−0.0004057, −0.0097804, 0.9999521	−0.0003794, −0.0097122, 0.9999528
Inclination azimuth of the tower central axis β	1 (RANSAC)	90°01′54″	90°01′05″	90°03′03″	90°01′25″
	2 (Marching Square)	90°03′02″	90°01′55″	90°01′24″	90°01′18″
Inclination angle of the tower central axis φ	1 (RANSAC)	0°29′30′′	0°30′12″	0°30′20″	0°29′17″
	2 (Marching Square)	0°30′13″	0°31′13″	0°33′39″	0°33′25″
Absolute linear displacement (m)	1 (RANSAC)	0.2470	0.2528	0.2539	0.2452
	2 (Marching Square)	0.2530	0.2613	0.2817	0.2798
Inclination and its mean value (‰)	1 (RANSAC)	8.58	8.78	8.82	8.52
		8.68			
	2 (Marching Square)	8.79	9.08	9.79	9.72
		9.35			
Mean deviation of inclination values (‰)	1 (RANSAC)	0.10	0.10	0.14	0.16
		0.12			
	2 (Marching Square)	0.56	0.27	0.44	0.37
		0.41			

## Data Availability

The data presented in this study are available in the article. Further inquiries can be directed to the corresponding author.
